# Dihydroartemisinin Unravels Dose-Dependent Transcriptomic Networks Orchestrating Ferroptosis and Metabolic Reprogramming in Colorectal Cancer

**DOI:** 10.3390/cimb48040342

**Published:** 2026-03-25

**Authors:** Zhaodi Zheng, Xitan Hou, Wenjuan Li, Leilei Zhang

**Affiliations:** College of Medical Imaging and Laboratory, Jining Medical University, Jining 272067, China

**Keywords:** Dihydroartemisinin (DHA), colorectal cancer, dose-dependent, transcriptomic profiling, ferroptosis, metabolic reprogramming

## Abstract

Background/Objectives: Dihydroartemisinin (DHA), a bioactive metabolite of *Artemisia annua*, displays potent antitumor activity in multiple cancers. However, its dose-dependent transcriptional regulatory networks in colorectal cancer (CRC) remain insufficiently understood. This study aimed to clarify the molecular mechanisms of low- and high-dose DHA in human CRC cells and reveal the dose-dependent crosstalk among related biological processes. Methods: We integrated RNA-seq transcriptomic profiling and functional validation in HCT116 cells treated with 20 μM (low-dose) or 50 μM (high-dose) DHA. Differentially expressed genes (DEGs) were screened at FDR ≤ 0.05 and |log_2_(fold change)| ≥ 1, followed by GO and KEGG enrichment analyses. Results: DHA inhibited cell viability dose-dependently, with an IC_50_ of 50 μM. We identified 280 and 678 DEGs in low-and high-dose groups, respectively. Low-dose DHA induced apoptosis via *GADD45α/β* and *ATF4/DDIT3*-mediated endoplasmic reticulum stress and triggered senescence through G2/M phase arrest. High-dose DHA mainly modulated gene expression signatures associated with ferroptosis by regulating iron homeostasis and lipid peroxidation at the transcriptional level. Both doses suppressed glycolysis, lipid, and folate metabolism; high-dose DHA also inhibited *MGAT5B*-mediated glycosylation. DHA regulated five core signaling pathways dose-dependently, with high-dose DHA further repressing *Wnt3a/16* and *BMP4/6*. Conclusions: This study first identifies ferroptosis-related gene networks as key transcriptional targets. It reveals dose-dependent crosstalk among cell death, senescence, metabolic reprogramming, and signaling, providing a transcriptomic framework and gene targets for optimizing DHA-based colorectal cancer therapy.

## 1. Introduction

Cancer is a disease characterized by the uncontrolled growth of abnormal cells. It ranks as the second leading cause of global morbidity and mortality, following cardiovascular diseases, posing a major threat to human health and survival. According to the latest estimates from the International Agency for Research on Cancer (IARC), approximately 20 million new cancer cases were diagnosed and about 9.7 million cancer-related deaths occurred worldwide in 2022. The data suggest that about one in five people will be diagnosed with cancer in their lifetime. However, mortality risk differs by sex: approximately one in nine men and one in twelve women are expected to die from the disease [1].

Colorectal cancer (CRC) stands as the third most frequently diagnosed malignancy and the second leading cause of cancer-related death globally. In 2022 alone, over 1.9 million new CRC cases and 904,000 CRC-associated deaths were documented, accounting for nearly 10% of total cancer incidence and mortality worldwide. Despite advances in chemotherapy, radiotherapy, targeted therapy and immunotherapy, the prognosis of advanced CRC remains dismal, largely attributed to late diagnosis, limited therapeutic efficacy against metastatic lesions, and the lack of precise targeted strategies. Thus, exploring novel high-efficiency, low-toxicity anti-tumor agents and deciphering their underlying molecular mechanisms is urgently needed to optimize CRC treatment regimens [1,2].

Natural compounds have emerged as promising sources of anti-cancer drugs due to their favorable safety profiles and multi-target regulatory properties. Dihydroartemisinin (DHA), the primary active metabolite of artemisinin extracted from the traditional Chinese medicinal herb *Artemisia annua*, is a well-established antimalarial agent. Accumulating evidence has uncovered its extra-antimalarial pharmacological activities, including anti-inflammatory, anti-pulmonary fibrotic and antioxidant effects. Notably, DHA exhibits potent anti-tumor activity against diverse malignancies, including CRC, pancreatic cancer, ovarian cancer and breast cancer, making it a hotspot in anti-cancer research [3,4]. A critical characteristic of DHA’s anti-tumor action is its dose-dependent effect, with low and high doses triggering distinct biological responses and molecular pathways in tumor cells.

Current studies have preliminarily explored the dose-dependent anti-cancer mechanisms of DHA in multiple tumor types, yet most findings are tumor-specific and lack consistency in CRC. Existing reports focus on single-dose DHA-mediated effects in CRC, such as apoptosis induction, migration inhibition and cell cycle arrest, while comprehensive investigations into the dose-specific transcriptional regulatory networks remain scarce. For instance, low-dose DHA modulates autophagy and cell motility in other cancers via distinct signaling axes [5,6,7]. For example, DHA (10–20 μM) suppresses cell proliferation, invasion, and migration in gastric cancer cells [8]. Furthermore, DHA-based nanodrug delivery systems (5–20 μM) downregulate reactive oxygen species modulator 1 (ROMO1) in ovarian cancer cells, thereby reducing intracellular ROS levels and inducing dose-dependent apoptosis [9]. Conversely, high-dose DHA elicits robust cytotoxicity by regulating apoptosis, angiogenesis and other pathways [10,11,12]. And at even higher doses, DHA (100–200 μM) inhibits bladder cancer cell migration and invasion by downregulating KDM3A and upregulating p21 expression [13]. Yet these dose-dependent mechanisms have not been systematically validated or elucidated in CRC cells. Particularly, the differential transcriptional profiles, crosstalk between cell death programs (apoptosis, ferroptosis) and metabolic reprogramming induced by low- and high-dose DHA in CRC remain largely unknown.

This knowledge gap severely hinders the rational optimization of DHA-based CRC therapy and the development of dose-adaptive therapeutic strategies. Therefore, this study utilized transcriptomic sequencing combined with functional assays to investigate the dose-dependent effects of low- and high-dose DHA in HCT116 CRC cells, screened differentially expressed genes and enriched signaling pathways, and clarified the distinct molecular mechanisms underlying low- and high-dose DHA intervention. This study aims to provide a transcriptomic foundation and novel gene targets for advancing DHA as a targeted therapeutic agent against CRC.

## 2. Materials and Methods

### 2.1. Cell Culture and Reagents

The HCT116 cell line was authenticated and tested for contamination, and purchased from the American Type Culture Collection (ATCC, Manassas, VA, USA). HCT116 cells were maintained in DMEM medium supplemented with 10% FBS and 1% penicillin/streptomycin sulfate (P1400; Solarbio, Beijing, China). Cells were cultured at 37 °C under an atmosphere of 5% CO_2_ incubator. Dihydroartemisinin (DHA; Sigma-Aldrich, St. Louis, MO, USA) was prepared as a sterile stock solution for subsequent experiments. Briefly, DHA powder was accurately weighed and dissolved in dimethyl sulfoxide (DMSO) to prepare a 0.2 mM master standard stock solution. The stock solution was thoroughly mixed to ensure complete solubilization, filtered through a 0.22 μm sterile filter to avoid microbial contamination, and stored at −80 °C in single-use aliquots to prevent repeated freeze–thaw cycles. Immediately before cell treatment, the DHA stock solution was diluted to the required working concentrations (10, 20, 30, 40, 50, 60, 70 and 80 μM) using pre-warmed DMEM complete medium containing 10% FBS, and the final DMSO concentration in all treatment groups was controlled to ≤0.1% to avoid solvent-induced cytotoxicity.

### 2.2. MTT Assay

Cell viability was assessed using the MTT assay (Solarbio, Beijing, China). Briefly, cells were seeded in 96-well plates at a density of 4000 cells per well and treated with varying doses (0, 10, 20, 30, 40, 50, 60, 70 and 80 μM) of DHA (Sigma-Aldrich, St. Louis, MO, USA) for 24 h. Following treatment, 20 μL of MTT solution (5 mg/mL) was added to each well, and the plates were incubated for 4 h. The medium was then carefully aspirated, and the formazan crystals were dissolved in 150 μL of DMSO (Solarbio, Beijing, China). Absorbance was measured at 490 nm using a microplate reader (Awareness, Palm City, FL, USA).

### 2.3. Colony Formation

Cells were plated in 6-well plates at a density of 1000 cells per well. After 24 h of culture, they were treated with different doses of DHA (0, 10, 20 μM) for 10 days to form colonies. Following treatment, the colonies were washed twice with ice-cold PBS, fixed with methanol for 20 min, and then stained with Crystal Violet Staining Solution (Beyotime, Shanghai, China) for 10 min after two washes with distilled water. Finally, images were captured using a digital camera.

### 2.4. SA-β-Gal Staining

Following the kit instructions (SA-β-gal staining kit, CST, Chicago, IL, USA), cells in 6-well plates were treated with DHA (50 μM, 24 h) or DHA (20 μM, 7 d). After PBS washes and fixation, cells were stained overnight at 37 °C (without CO_2_) and then imaged using an inverted bright-field microscope (Olympus, Tokyo, Japan) with a digital camera.

### 2.5. RNA Extraction, Illumina Sequencing, and Alignment Using Reference Genome

Total RNA was extracted from HCT116 cells treated with 20 μM and 50 μM DHA using RNAiso Plus (TaKaRa, Dalian, China), according to the manufacturer’s instructions. RNA quality and purity were assessed by electrophoresis on 1.2% agarose gels and quantified using a NanoDrop 2000 spectrophotometer (Thermo Fisher Scientific, Waltham, MA, USA).

For RNA-Seq library preparation, 5 μg of total RNA per sample was used. Polyadenylated mRNA was enriched using oligo-dT beads (Qiagen, Hilden, Germany). The enriched mRNA was fragmented by incubation with fragmentation buffer, followed by first-strand cDNA synthesis with random primers. Second-strand cDNA was then generated using dNTPs, RNase H, and DNA polymerase I. cDNA fragments were purified with the QiaQuick PCR extraction kit (Qiagen, Venlo, The Netherlands). Subsequent steps included end repair, poly(A) tailing, and ligation of Illumina sequencing adapters. The final libraries were amplified by PCR and subjected to paired-end sequencing on an Illumina HiSeq2500 platform (Gene Denovo Biotechnology Co., Guangzhou, China). Raw sequencing reads were processed with fastp version 1.0 [14] to obtain high-quality clean reads. The reference genome was indexed, and paired-end clean reads were aligned to it using HISAT2 version 2.4 [15].

### 2.6. Differentially Expressed Genes (DEGs) and Bioinformatics Analysis

Gene expression levels were calculated as fragments per kilobase of transcript per million mapped reads (FPKM) for quantification and visualization. Differential expression analysis was carried out using the DESeq2 software version 1.46.0 [16] for the following comparisons: H116_DHAC vs. H116DHA1, H116_DHAC vs. H116DHA2, and H116DHA1 vs. H116DHA2. Genes with an absolute |log_2_(fold change)|, a *p*-value < 0.05, and a false discovery rate (FDR) ≤ 0.05 were considered differentially expressed genes (DEGs).

Functional annotation of the DEGs was conducted through Gene Ontology (GO) enrichment analysis using the Blast2GO software version 2.5 [17] and Kyoto Encyclopedia of Genes and Genomes (KEGG) pathway analysis via the KAAS server version 2.1 [18]. Finally, hierarchical clustering of DEGs based on FPKM values was visualized using an online analysis tool (https://www.omicshare.com/tools/, accessed on 15 December 2025).

### 2.7. Drawings and Statistical Analysis

Figures were prepared and assembled using Adobe Photoshop CC and Adobe Illustrator CC (San Jose, CA, USA). Data are expressed as the mean ± standard deviation (SD). Statistical analysis was performed using Prism software (version 8.0), including regression fitting and Student’s *t*-test. Significance levels are denoted by asterisks (* *p* < 0.05, ** *p* < 0.01).

## 3. Results

### 3.1. DHA Inhibited Proliferation of Colorectal Cancer HCT116 Cells in a Dose-Dependent Manner

**Figure 1:**
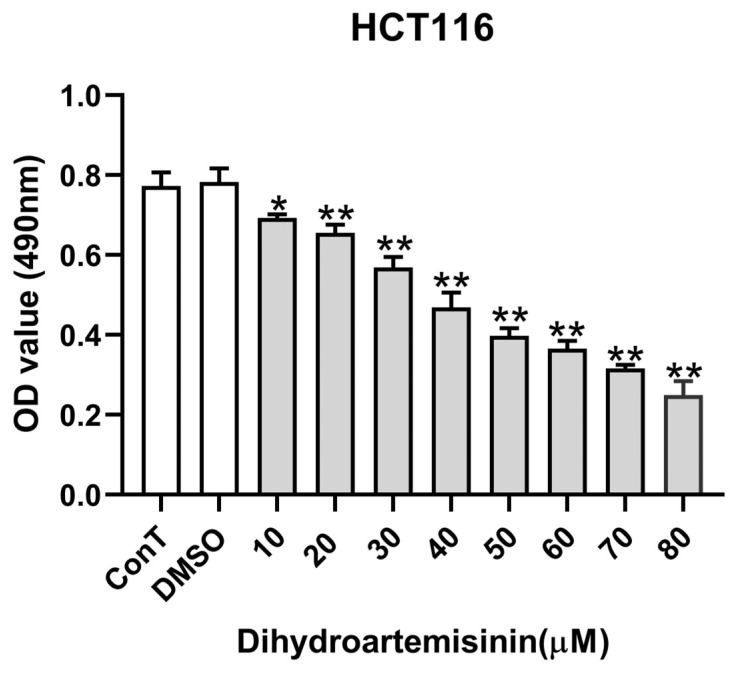
Dose-dependent effects of DHA on viability of HCT116 cells. Significant suppression of cell viability following 24 h treatment with increasing DHA concentrations (0–80 μM). White bars represent control (ConT) and DMSO vehicle-treated groups, while gray bars indicate DHA-treated groups. Data are presented as mean ± SD from *n* = 3 independent biological replicates. Statistical analysis was performed using one-way ANOVA followed by Tukey’s post hoc test compared to the control group. * *p* < 0.05, ** *p* < 0.01, vs. vehicle control (0 μM).

Previous studies from our laboratory demonstrated that DHA suppresses cell proliferation and cell migration of human breast and liver cancer cells [3]. In this study, we first confirmed DHA’s effect on cellular viability through MTT assays. HCT116 cells were treated with increasing doses of DHA (0, 10, 20, 30, 40, 50, 60, 70, 80 μM) for 24 h. Results revealed dose-dependent inhibition of HCT116 cell viability (IC_50_ = 50 μM), with 20 μM DHA reducing viability by 20–30% (Figure 1). Based on these MTT results, 20 μM was defined as a low dose with mild cytotoxicity (≈20–30% inhibition), while 50 μM was set as a high dose corresponding to the IC_50_ value. This dose design allowed us to investigate distinct dose-dependent transcriptional responses underlying DHA-induced growth inhibition.

### 3.2. Illumina Sequencing and Quality Assessment

The cDNA libraries derived from 9 groups at three concentrations treatment (0, 20 and 50 μM) were constructed and sequenced using the Illumina deep-sequencing platform to identify DHA responding to HCT116 cells. A total of 408.93 Mb raw reads were generated, and 407.66 Mb clean reads were obtained with an average Q30 of 94.11% and 94.42%, respectively (Table 1). The high-quality clean reads were mapped to the reference Human genome (total mapping rates > 94.69%) (Table 1). PCA (Figure S1) and the Inter-sample correlation heatmap (Figure S2) revealed that Samples from the control, 20 μM DHA, and 50 μM DHA groups formed distinct clusters, demonstrating clear separation of transcriptomic profiles and good reproducibility within each group. All RNA-Seq data were submitted to the NCBI Sequence Read Archive (accession number: PRJNA1428755).

### 3.3. Transcriptome Changes and Pathway Enrichment in DHA-Treated HCT116 Cells

**Figure 2:**
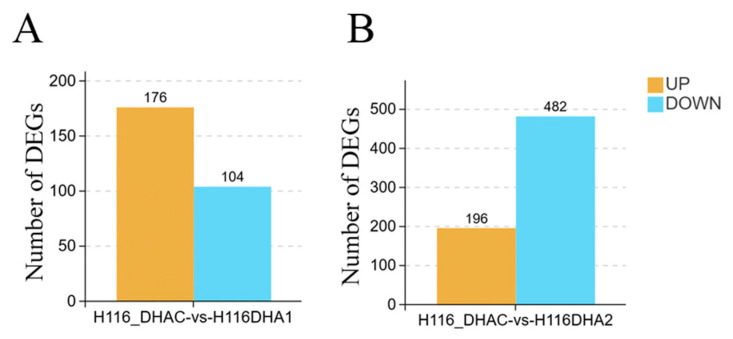
The number of DEGs in cancer cells transcriptome via a pairwise comparison. (**A**) 20 μM DHA treatment (H116_DHA1 vs. control) yielded 280 DEGs (176 upregulated, 104 downregulated). (**B**) 50 μM DHA treatment (H116_DHA2 vs. control) yielded 678 DEGs (196 upregulated, 482 downregulated). Color codes: orange, upregulated genes; light blue, downregulated genes.

Transcriptomic profiling of HCT116 cells treated with 20 μM (H116DHA1) or 50 μM (H116DHA2) DHA for 24 h was performed using RNA sequencing. Differential gene expression analysis was conducted with DEseq2 software [16] comparing three sample pairs: H116_DHAC vs. H116DHA1, H116_DHAC vs. H116DHA2, H116DHA1 vs. H116DHA2. The criteria for DEGs were set as FDR ≤ 0.05 and log_2_(fold change) ≥ 1, and the DEGs were screened and identified. We treated HCT116 cells with DHA (20 μM and 50 μM) for 24 h and evaluated the transcriptional changes through RNA sequencing analysis. The results of transcriptome analysis showed that 20 μM DHA produced 280 DEGs, among which 176 genes were upregulated and 104 genes were downregulated. High-dose 50 μM DHA generated 678 DEGs, among which 196 genes were upregulated and 482 genes are downregulated. Compared with the H116DHA1 (20 μM) group, the H116DHA2 (50 μM) group obtained more differential genes (398 genes) in cancer cells (FDR ≤ 0.05, log_2_FC ≥ 1; Figure 2); it was initially believed that low and high doses may affect different signaling pathways and mechanisms of action within CRC cells, and the cancer cells require a higher dose to trigger intracellular programs.

#### 3.3.1. GO Analysis

To investigate the impact of DEGs on HCT116 cells treated with 20 μM DHA, a comparison with the GO database revealed that a total of 4001 unigenes were annotated. These unigenes were categorized into three primary GO categories—biological processes, molecular functions, and cellular components—as well as 55 secondary GO categories (Figure S3). Within the primary category of biological processes, “cellular process” was the most enriched term, containing 251 unigenes, which accounted for 6.27% of all GO-annotated unigenes. Other significantly represented terms included “biological regulation” (209 unigenes, 5.2%), “regulation of biological processes” (199 unigenes, 4.97%), “metabolic processes” (189 unigenes, 4.72%), “response to stimulus” (183 unigenes, 4.57%), and “Signaling” (38 unigenes, 3.45%). Under the molecular function category, “binding” was the most abundant term, comprising 235 unigenes (5.87%), followed by “catalytic activity” with 101 unigenes (2.52%). In the cellular component category, “cell” contained 243 unigenes (6.07%), “cell part” had 243 unigenes (6.07%), “organelle” included 201 unigenes (5.02%), and “organelle part” contained 129 unigenes (3.22%).

To elucidate the role of DEGs in the response of HCT116 cells to 50 μM DHA, comparison with the Gene Ontology (GO) database resulted in the annotation of 9706 unigenes. These unigenes were distributed across the three primary GO categories—Biological Process, Molecular Function, and Cellular Component—and 56 secondary GO categories (Figure S4). Based on the assigned GO terms, enrichment analysis revealed the following under the primary categories: within Biological Process, “cellular process” was enriched with 577 unigenes (5.94% of total annotated unigenes), “Biological regulation” with 481 unigenes (4.96%), “Regulation of biological process” with 458 unigenes (4.72%), “metabolic process” with 408 unigenes (4.20%), and “Response to stimulus” with 408 unigenes (4.20%); under Molecular Function, “Binding” was enriched with 497 unigenes (5.12%) and “Catalytic activity” with 201 unigenes (2.07%); within Cellular Component, “Cell” contained 579 unigenes (5.96%), “Cell part” 579 unigenes (5.96%), “Organelle” 460 unigenes (4.74%), and “Organelle part” 309 unigenes (3.18%).

The results show that 50 μM DHA treatment annotated 5705 more unigenes than 20 μM DHA. Both treatments were predominantly represented in the primary categories of Biological Processes, including “cellular process,” “biological regulation,” “regulation of biological process,” and “metabolic process.” Additionally, the molecular function category was mainly enriched in “binding,” while the cellular component category primarily included “cell,” “cell part,” “organelle,” and “organelle part”.

#### 3.3.2. KEGG Analysis

KEGG analysis is commonly employed as a powerful tool to identify cellular signaling pathways associated with unigene function. In this study, a total of 495 unigenes from samples treated with 20 μM DHA were annotated within specific categories in the KEGG database (Figure S5). The category containing the highest number of unigenes was “signal transduction” (58 unigenes), accounting for 11.72% of all KEGG-annotated unigenes. This was followed by “global and overview” (37 unigenes, 7.47%), “cancer overview” (35 unigenes, 7.07%), and “infectious disease: viral” (29 unigenes, 5.86%), and “immune system” (27 unigenes, 5.45%).

A total of 925 unigenes in the 50 μM DHA treatment group were assigned to specific categories in the KEGG database (Figure S6), among which the category with the largest number was “signal transduction” (106 unigenes, accounting for 11.64% of the total KEGG annotated unigenes). Secondly, there were “Global and overview maps” (61 unigenes, accounting for 6.59%) and “infectious disease: “viral” (57 unigenes, accounting for 6.16%) and “cancer overview” (57 unigenes, accounting for 6.16%).

The results showed that the 50 μM DHA treatment group annotated more unigenes in the KEGG database than the 20 μM DHA treatment group. Both treatments were mainly enriched in the “signal transduction” and “cancer overview” pathways.

### 3.4. DHA Regulated the Cell Death of CRC Cells

**Figure 3:**
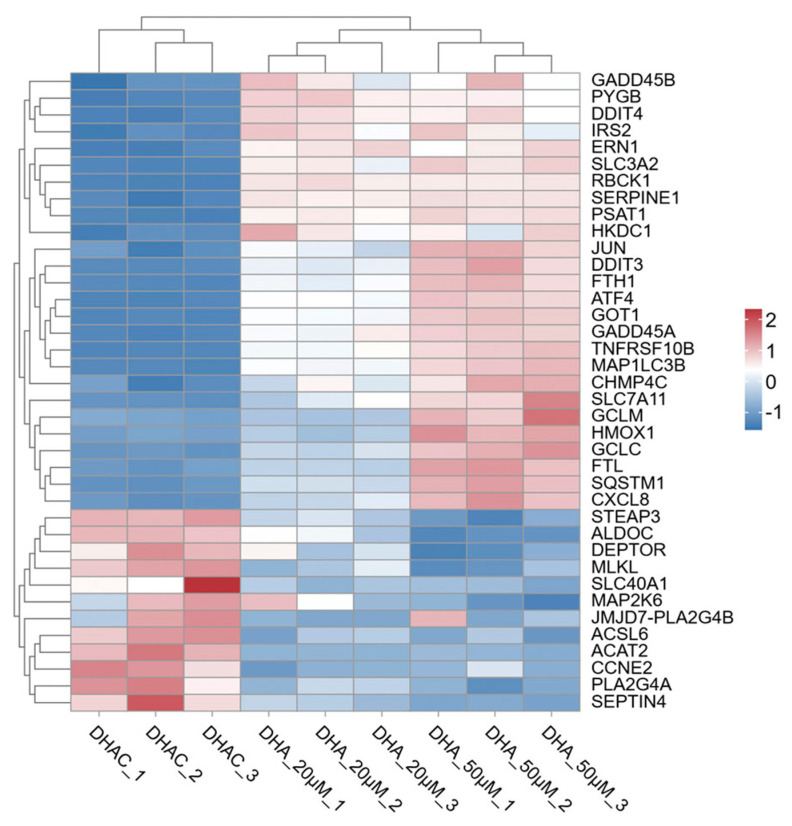
Hierarchical clustering of DEGs in regulating cell death. Gene expression levels are color-coded on a continuum from low (navy) to medium (white) and high (firebrick). Color scale: blue, downregulated gene expression; red, upregulated gene expression; white, no change.

Numerous studies have demonstrated that DHA plays a significant role in anti-malarial, anti-inflammatory, antioxidant, and chemopreventive activities. In recent years, it has also been found to exhibit promising preventive and therapeutic effects against cancer [3]. Therefore, we performed a hierarchical clustering analysis of the DEGs involved in pathways related to the anti-cancer effects of DHA (Figure 3).

#### 3.4.1. Apoptosis Pathway of Cancer Cells

**Figure 4:**
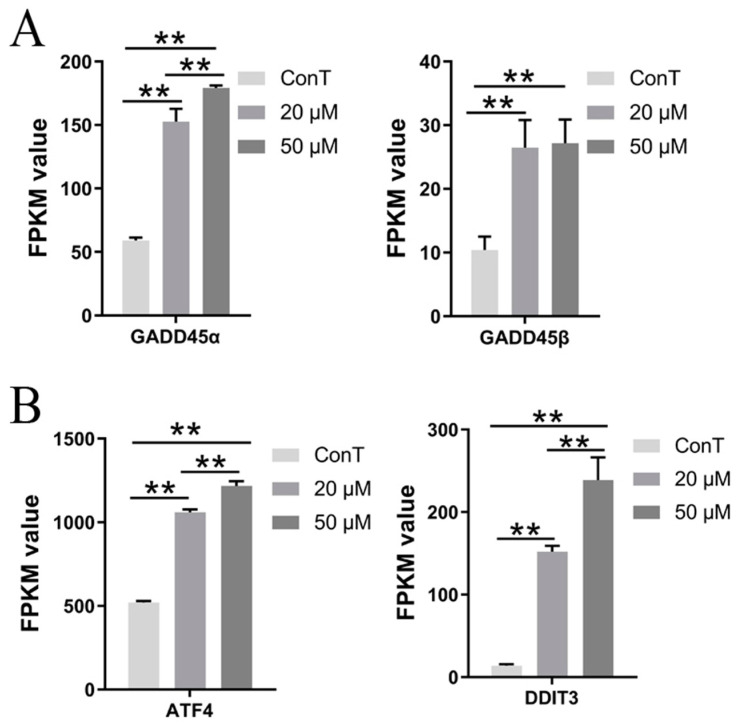
Transcriptome expression levels of (**A**) *GADD45α/GADD45β* and (**B**) *ATF4/DDIT3*. Data are presented as mean ± SD from *n* = 3 independent biological replicates. Statistical analysis was performed using one-way ANOVA followed by Tukey’s post hoc test compared to the control group. ** *p* < 0.01.

In the transcriptome of HCT116 cells treated with 20 and 50 μM DHA, a total of 8 DEGs related to apoptosis were identified. The GADD45 family comprises genes whose expression was induced under conditions of DNA damage, growth arrest and cell apoptosis. This family includes three members—GADD45α, GADD45β, and GADD45γ—which play important roles in the cellular response to external stress [19]. DHA upregulated the expression of *GADD45α* and *GADD45β*, and the upregulation of *GADD45α* was dose-dependent (Figure 4A). These proteins can interact with multiple cellular signaling pathways, induce cell cycle arrest, promote DNA repair, and initiate apoptosis, thereby maintaining genomic stability and preventing malignant transformation.

Furthermore, endoplasmic reticulum stress (ERS) contributes significantly to tumor progression. ATF4 (activating transcription factor 4) and DDIT3 (DNA damage-inducible transcript 3, also known as CHOP) are two ERS-related genes involved in cell cycle arrest and the regulation of apoptotic signaling molecules [20]. DHA upregulated the expression of *ATF4* and *DDIT3* in a dose-dependent manner, suggesting its regulatory role in the molecular mechanisms underlying tumor cell apoptosis (Figure 4B).

#### 3.4.2. Necrotic Apoptotic Pathway

**Figure 5:**
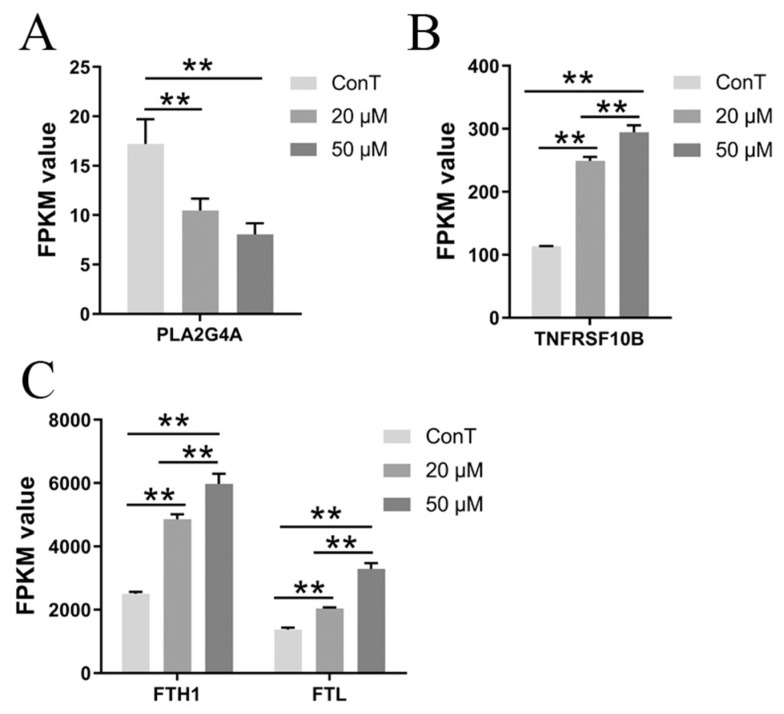
Transcriptome expression levels of (**A**) *PLA2G4A*, (**B**) *TNFRSF10B* and (**C**) *FTH1/FTL*. Data are presented as mean ± SD from *n* = 3 independent biological replicates. Statistical analysis was performed using one-way ANOVA followed by Tukey’s post hoc test and two-way ANOVA compared to the control group. ** *p* < 0.01.

In the transcriptome of HCT116 cells treated with DHA, a total of 9 differentially expressed genes (DEGs) associated with cell necrosis were identified. Among these, PLA2G4A, which encodes the cytosolic phospholipase A2 alpha (cPLA2α), is a key enzyme in arachidonic acid metabolism that plays a pivotal role in regulating cell death [21]. DHA treatment markedly reduced *PLA2G4A* expression and promoted cancer cell death, although this effect did not exhibit clear dose dependence (Figure 5A). Furthermore, TNFRSF10B—a TRAIL receptor belonging to the TNF receptor superfamily located on the cell membrane—binds to its ligand TRAIL and forms trimers, thereby assembling the death-inducing signaling complex (DISC) to initiate cell death [22]. DHA increased *TNFRSF10B* expression, ultimately inducing death in CRC cells (Figure 5B). Moreover, a higher dose of DHA was more effective than lower dose in modulating genes associated with necroptosis pathways and exerting anti-cancer effects. In addition, DHA also dose-dependently elevated ferritin genes (*FTH1* and *FTL*) to enhance oxidative stress and activate necrosis-related pathways, leading to increased release of iron ions (Figure 5C).

#### 3.4.3. DHA Induced Ferroptosis Pathways in CRC Cells

**Figure 6:**
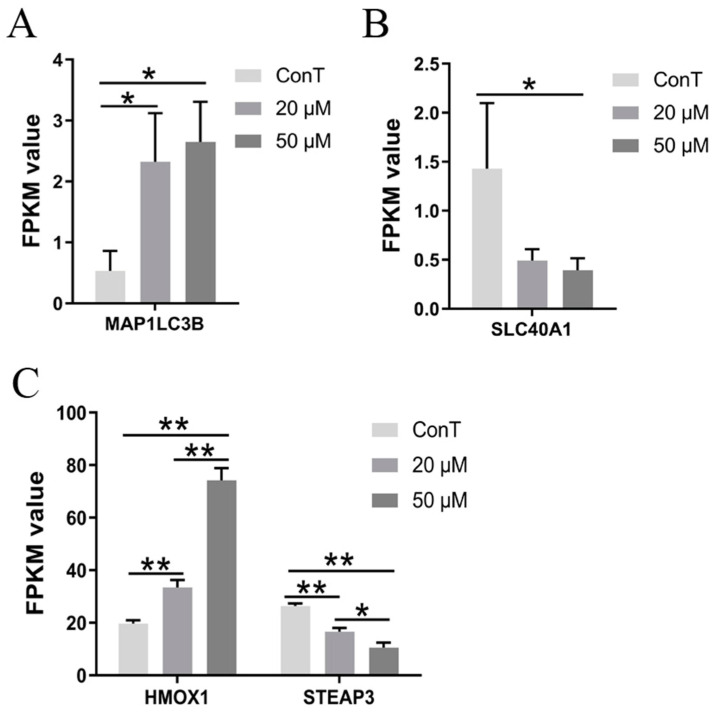
Transcriptome expression levels of genes related to the ferroptosis pathway ((**A**) *MAP1LC3B*, (**B**) *SLC40A1*, (**C**) *HMOX1* and *STEAP3*). Data are presented as mean ± SD from *n* = 3 independent biological replicates. Statistical analysis was performed using one-way ANOVA followed by Tukey’s post hoc test and two-way ANOVA compared to the control group. * *p* < 0.05, ** *p* < 0.01.

A total of 11 DEGs associated with ferroptosis were identified in the transcriptome of HCT116 cells treated with 50 μM DHA. Ferroptosis is a form of regulated cell death driven by iron-dependent lipid peroxidation. Microtubule-associated protein 1 light chain 3B (MAP1LC3B, also known as ATG8) is involved in autophagosome formation and plays a key role in cellular autophagy. Autophagy can elevate intracellular iron levels through ferritin degradation, promoting the accumulation of reactive oxygen species (ROS) and ultimately leading to ferroptosis [23]. Our transcriptomic data indicated that 20 and 50 μM DHA upregulated *MAP1LC3B* levels, suggesting a potential role of ferroptosis in DHA-mediated cell death (Figure 6A). Solute carrier family 40 member 1 (SLC40A1) is crucial for exporting iron from the intracellular to the extracellular environment. Dysregulation of SLC40A1 expression disrupts cellular iron homeostasis, resulting in intracellular iron overload and subsequent ferroptosis [24]. Only a high dose of DHA decreased *SLC40A1* gene expression to promote ferroptosis (Figure 6B).

HMOX1 (heme oxygenase 1) plays an important role in the cellular response to oxidative stress and inflammation, primarily by catalyzing the degradation of heme into biliverdin, carbon monoxide, and ferrous iron (Fe^2+^). The metalloreductase STEAP3, which is highly expressed in various cancers, is significantly associated with poor patient prognosis. Downregulation of STEAP3 has been shown to increase levels of ROS and malondialdehyde while reducing glutathione and superoxide dismutase in ovarian cancer cells [25]. In our study, DHA upregulated HMOX1 expression and inhibited STEAP3 expression in a dose-dependent manner, suggesting that ferroptosis may be involved in DHA-induced cell death in CRC cells (Figure 6C). Furthermore, high-dose DHA also intensified oxidative stress by upregulating ferritin genes (*FTH1* and *FTL*) (Figure 5C), promoting iron ion release and thereby contributing to necrosis-related pathways.

### 3.5. DHA Induced Aging of Cancer Cells

#### 3.5.1. The Effects of DHA on Clone Formation and the Induction of Aging in HCT116 Cells

**Figure 7:**
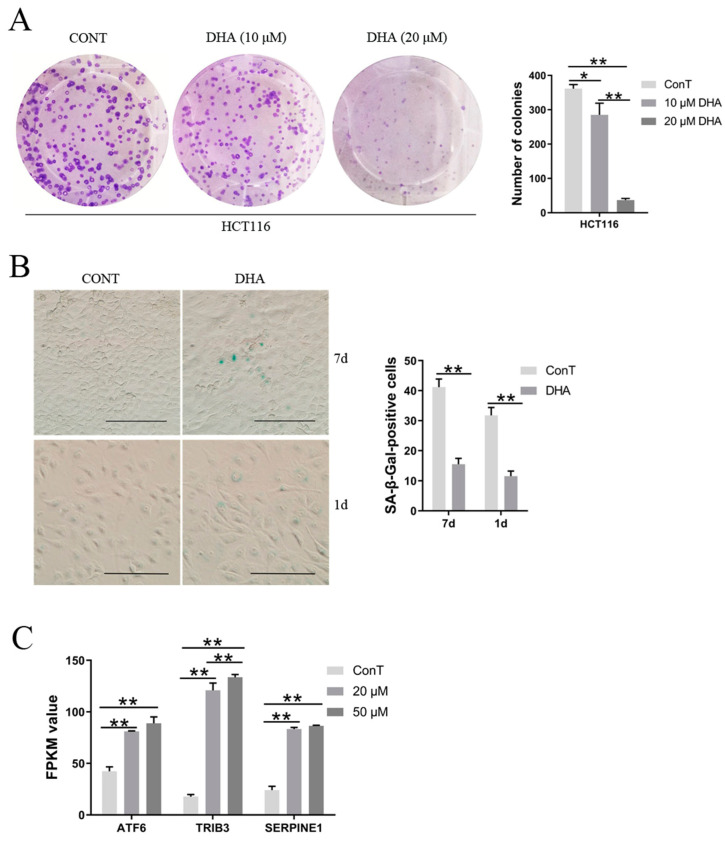
(**A**) The effect of DHA on the induction of the colony formation in cancer cells. (**B**) The SA-β-GAL staining results of HCT116 cells after exposure to 20 μM DHA for 7 days or 50 μM DHA for 1 day. The scale is 10 μm. (**C**) Transcriptome expression levels of genes related to the aging pathway. Data are presented as mean ± SD from *n* = 3 independent biological replicates. Statistical analysis was performed using one-way ANOVA followed by Tukey’s post hoc test and two-way ANOVA compared to the control group. * *p* < 0.05, ** *p* < 0.01. Light gray bars: Control group (ConT); Medium gray bars: 10 μM or 20 μM DHA treatment group; Dark gray bars: 50 μM DHA treatment group.

We first evaluated the cytotoxic effect of DHA on HCT116 cancer cells using a colony formation assay. As shown in Figure 7A, DHA significantly reduced colony numbers in a dose-dependent manner (285.30 colonies with 10 μM DHA and 36.98 colonies with 20 μM DHA, compared with 361.60 colonies in the control group). Next, we examined whether DHA induces cellular senescence in cancer cells. After treatment with 20 μM DHA for 7 days, HCT116 cells exhibited flattened and enlarged morphology, characteristics of senescent cells, whereas no such changes were observed in the control group, suggesting that DHA triggers cellular senescence. To confirm this, SA-β-Gal staining was performed. Quantitative analysis showed a significantly higher percentage of SA-β-Gal-positive cells in the 20 μM DHA-treated group (41.17% in HCT116) compared with the control group (15.57%) (Figure 7B). To shorten the experimental timeframe, additional SA-β-Gal staining was carried out following 50 μM DHA treatment for 1 day. Under these conditions, DHA-treated cells still showed a marked increase in SA-β-Gal-positive cells (31.79% in HCT116) relative to the control (11.55%) (Figure 7B). Together, these results demonstrate that DHA induces cellular senescence and suppresses colony formation in HCT116 cancer cells.

#### 3.5.2. DHA Regulated the Expression of Genes Related to Aging in Cancer Cells

DNA damage and mitochondrial dysfunction can trigger an ERS response, which in turn promotes the aging of cancer cells. ERS-related genes such as ATF4/6, DDIT3, and TRIB3 (tribbles pseudokinase 3) are involved in cell cycle regulation [20]. DHA enhanced *ATF6* levels and the *ATF4/DDIT3/TRIB3* signaling pathway, resulting in cell cycle arrest at the G2/M phase and inducing senescence in cancer cells (Figure 4B and Figure 7C). DHA also upregulated the expression of *PAI1* (also known as *SERPINE1*) without a dose-dependent manner, a downstream target of p53, leading to DNA damage and subsequent senescence in HCT116 cells (Figure 7C). Furthermore, GADD45 is able to physically interact with Cdc2 to directly inhibit the activity of the Cdc2/Cyclin B1 complex, leading to G2 phase arrest and cellular senescence [19,26]. DHA elevated the expression of *GADD45*, thereby promoting senescence in CRC cells (Figure 4A). Notably, a higher dose of DHA (50 μM) exerted a more pronounced pro-senescence effect on cancer cells than a lower dose (20 μM).

### 3.6. DHA Regulated the Metabolic Process of Cancer Cells

**Figure 8:**
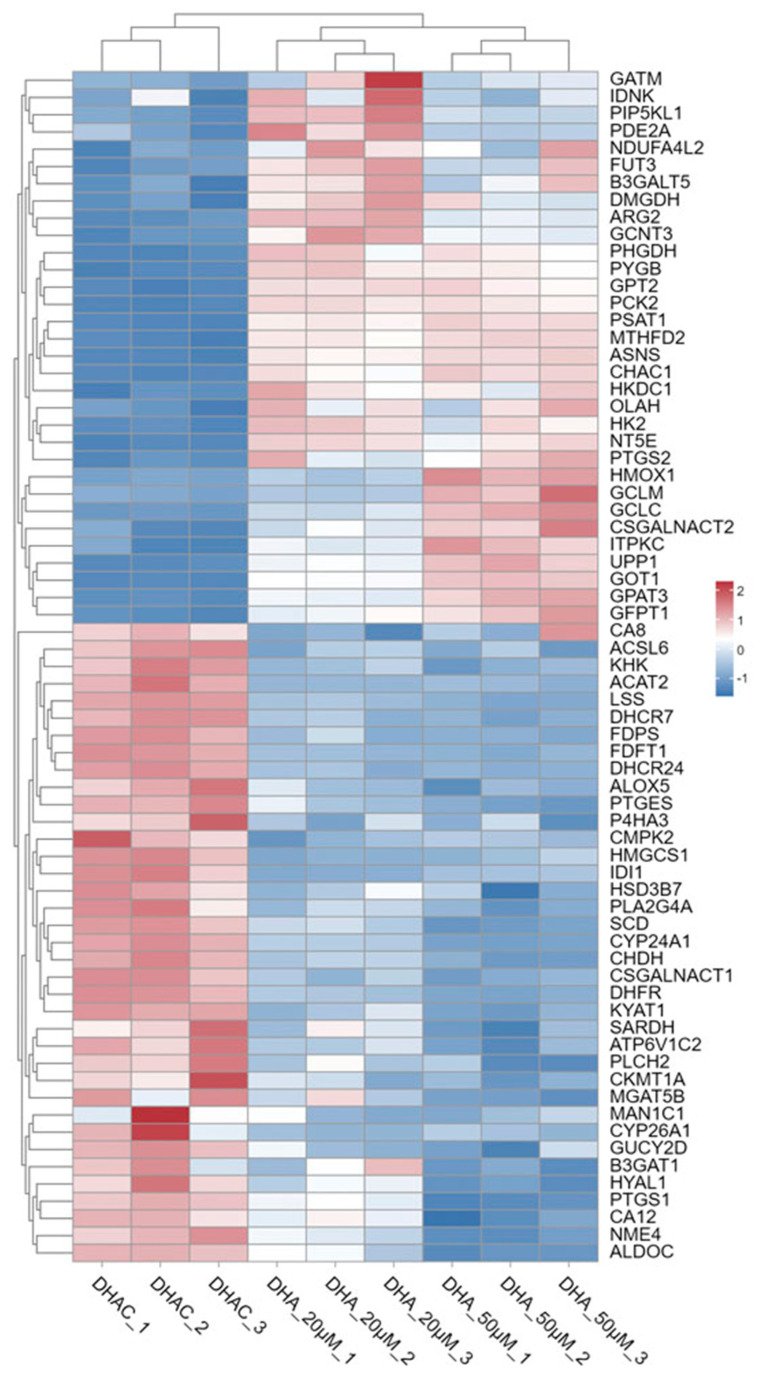
Hierarchical clustering analysis of DEGs in the metabolic pathway. Color scale: blue, downregulated gene expression; red, upregulated gene expression; white, no change.

As one of the core hallmarks of malignant tumors, metabolic reprogramming represents a critical metabolic rewiring event in cancer cells, which reconfigures intracellular metabolic pathways to meet the extraordinary demands for energy and biomacromolecule synthesis, thereby enabling cancer cells to achieve uncontrolled rapid proliferation, aggressive invasion of adjacent stromal tissues, and efficient distant metastasis [27]. Based on KEGG pathway enrichment analysis, we identified a total of 1478 genes primarily enriched in metabolism-related pathways. Among these, 37 differentially expressed genes (DEGs) were detected following treatment with 20 μM DHA, while 50 μM DHA treatment yielded 60 DEGs. Subsequently, hierarchical clustering analysis was performed on the DEGs involved in these pathways under different DHA doses (Figure 8).

**Figure 9:**
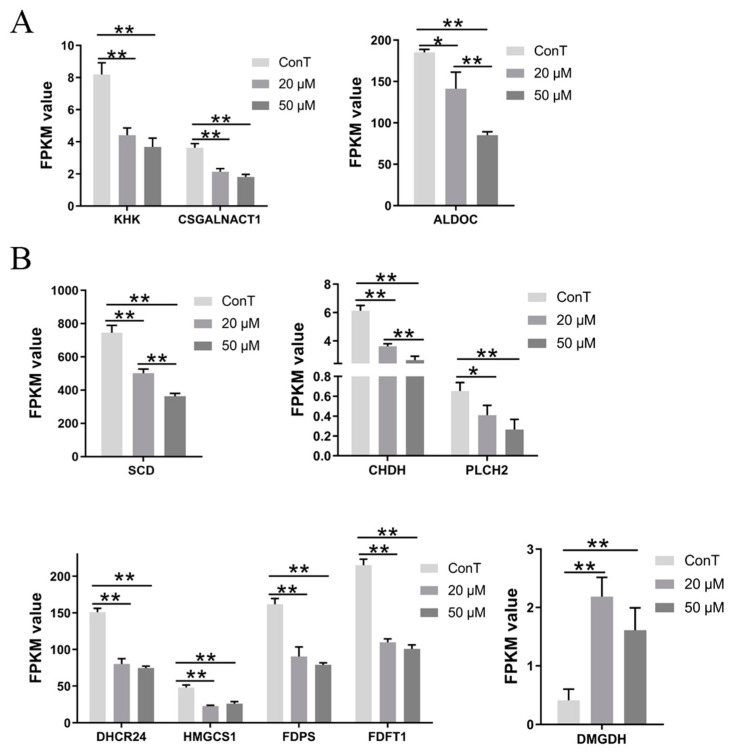
Expression of metabolic genes (**A**) *KHK*, *CSGALNACT1*, and *ALDOC*, (**B**) *SCD*, *CHDH*, *PLCH2*, *DHCR24*, *HMGCS1*, *FDPS*, *FDFT1*, and *DMGDH* upon DHA treatment in cancer cells. Data are presented as mean ± SD from *n* = 3 independent biological replicates. Statistical analysis was performed using one-way ANOVA followed by Tukey’s post hoc test and two-way ANOVA compared to the control group. * *p* < 0.05, ** *p* < 0.01.

Glycolysis is a central metabolic pathway that converts glucose into cellular energy. Fructokinase (KHK), the rate-limiting enzyme in fructose catabolism, acts as a serine/threonine protein kinase and promotes tumor growth, proliferation, and metastasis by activating multiple signaling pathways and metabolic processes [28]. CSGALNACT1 encodes chondroitin N-acetylgalactosaminyltransferase 1, an enzyme involved in glycosaminoglycan chain biosynthesis. Dysregulation of this biosynthetic pathway is linked to enhanced cancer invasiveness and metastasis [29]. Additionally, ALDOC (fructose-1,6-bisphosphate aldolase C) catalyzes the cleavage of fructose-1,6-bisphosphate into glyceraldehyde-3-phosphate and dihydroxyacetone phosphate, a key step in glycolysis. Members of the aldolase family support tumor cell proliferation by rewiring cellular metabolism [30]. Our findings indicate that DHA dose-independently downregulated the expression of *KHK*, *CSGALNACT1* and reduced *ALDOC* levels in a dose-dependent manner (Figure 9A).

To support their proliferation and growth, malignant tumors undergo corresponding adaptations in lipid metabolism. Stearoyl-CoA desaturase-1 (SCD1) is a key enzyme in fatty acid metabolism that converts saturated fatty acids into monounsaturated fatty acids. Elevated SCD1 expression was associated with poor prognosis in multiple cancer types. Interestingly, while SCD1 can promote an iron-dependent form of cell death known as ferroptosis, its upregulation may also protect cancer cells from ferroptotic stress [31]. Choline dehydrogenase (CHDH), a mitochondrial enzyme involved in choline metabolism and linked to mitophagy, is associated with the progression of several malignancies and may serve as a potential biomarker for colon adenocarcinoma. Dimethylglycine dehydrogenase (DMGDH), another enzyme in choline catabolism, has been shown to reduce the invasive and metastatic potential of liver cancer cells when highly expressed [32]. Phospholipase C eta 2 (PLCH2), a member of the phospholipase family, plays a key role in cellular signal transduction, particularly in lipid metabolism and related signaling. Dysregulated expression of PLCH2 is implicated in the development and progression of various cancers [33]. DHCR24 is a key enzyme in cholesterol biosynthesis, involved in the synthesis and regulation of cellular cholesterol. Its expression and activity significantly influence cholesterol production [34]. Hydroxy-3-methylglutaryl-CoA synthase 1 (HMGCS1), another key enzyme in the cholesterol synthesis pathway, catalyzes the condensation of two acetyl-CoA molecules to form HMG-CoA. HMG-CoA can be further metabolized into ketone bodies to supply cellular energy. HMGCS1 is upregulated in certain cancer cells, likely as part of metabolic reprogramming that supports tumor growth and dissemination [35]. Farnesyl pyrophosphate synthase (FDPS) functions in the mevalonate pathway. It is elevated in tumor cells to meet the increased demand for terpenoids and cholesterol required for rapid proliferation [36]. Farnesyl-diphosphate farnesyltransferase 1 (FDFT1) is a crucial enzyme in endogenous cholesterol synthesis and a reported direct target of artemisinin. It has been shown that artemisinin can induce breast cancer cell apoptosis by modulating FDFT1 and the NF-κB pathway [37]. Our transcriptomic data indicated that *SCD*, *CHDH*, *PLCH2*, *DHCR24*, *HMGCS1*, *FDPS*, and *FDFT1* were highly expressed in colorectal cancer cells. DHA treatment in a dose-dependent manner reduced *SCD1* and *CHDH* expression, decreased other genes and increased *DMGDH* levels in a dose-independent manner (Figure 9B), and modulated lipid-related metabolism, thereby suppressing CRC progression.

**Figure 10:**
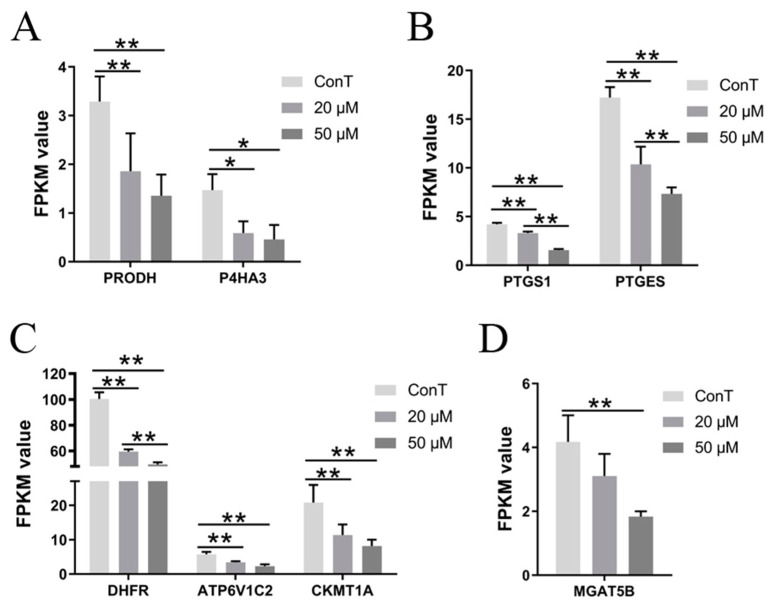
The levels of Metabolic genes (**A**) *ProDH* and *P4HA3*, (**B**) *PTGS1* and *PTGES*, (**C**) *DHFR*, *ATP6V1C2*, and *CKMT1A*, (**D**) *MGAT5B* treated with DHA of cancer cells. Data are presented as mean ± SD from *n* = 3 independent biological replicates. Statistical analysis was performed using one-way ANOVA followed by Tukey’s post hoc test and two-way ANOVA compared to the control group. * *p* < 0.05, ** *p* < 0.01.

Proline dehydrogenase (ProDH) is a key mitochondrial enzyme involved in proline catabolism, which contributes to cellular energy production. In some tumor cells, ProDH activity is abnormally elevated, a feature potentially linked to tumor metabolic reprogramming [38]. Prolyl 4-hydroxylase subunit alpha-3 (P4HA3) is a critical catalytic component in collagen synthesis and plays a significant role in the initiation, progression, and prognosis of various malignancies. Deficiency of P4HA3 has been shown to inhibit tumor cell proliferation, migration, and invasion [39]. Our results indicate that DHA prevented the progression of CRC by downregulating the expression of *ProDH* and *P4HA3* without showing clear dose dependence (Figure 10A).

Cyclooxygenase-1 (COX-1), encoded by the PTGS1 gene, is involved in arachidonic acid metabolism and functions as a key enzyme in the prostaglandin synthesis pathway. COX-1 modulates the phospholipid composition of the cell membrane, thereby influencing the transport and activation of apoptosis-related signaling molecules [40]. Prostaglandin E synthase (PTGES), another key enzyme in the arachidonic acid pathway, catalyzes PGE2 synthesis and was upregulated in pancreatic cancer, where it promotes tumor initiation and progression [41]. DHA treatment reduced *PTGS1* and *PTGES* gene levels in a dose dependent manner, further inhibiting prostaglandin synthesis and contributing to its anti-tumor effects (Figure 10B).

In addition, dihydrofolate reductase (DHFR) is a crucial enzyme in folate metabolism, responsible for reducing dihydrofolate to tetrahydrofolate—an essential cofactor in DNA synthesis and repair. Aberrant DHFR activity is closely associated with cancer development [42]. ATP6V1C2, a member of the V-type ATPase subunit gene family, participates in establishing and maintaining proton gradients, thereby driving multiple energy-dependent cellular processes. ATP6V1C2 was found to be markedly upregulated in colorectal cancer (CRC), and it has the potential to serve as a novel therapeutic target for CRC [43]. Mitochondrial creatine kinase 1A (CKMT1A) facilitates the interchange of phosphocreatine and creatine across mitochondrial membranes and plays an important role in cellular energy metabolism. It also drives the proliferation, migration, and invasive capacity of breast cancer cells [27]. In this study, DHA treatment downregulated the expression of *DHFR*, *ATP6V1C2*, and *CKMT1A* (Figure 10C), contributing to its anti-cancer effect. Among these genes, DHFR expression was dose-dependent. Furthermore, DHA promoted the expression of heme oxygenase 1 (*HMOX1*) (Figure 6C), the rate-limiting enzyme in heme catabolism, thereby impeding tumor cell progression.

Altered protein glycosylation is commonly observed in tumor cells. In particular, N-glycan branching catalyzed by MGAT5B represents a prevalent tumor-associated glycan modification. The increased branching exerts pro-tumor effects through multiple mechanisms, including modulation of cell adhesion and signaling [44]. Transcriptomic analysis revealed that *MGAT5B* was overexpressed in CRC cells, where it alters glycosylation patterns, promotes tumor cell adhesion and signaling, and facilitates tumor growth and dissemination. Notably, treatment with a high dose of DHA reduced *MGAT5B* expression, thereby suppressing cancer progression (Figure 10D).

### 3.7. Signaling Pathway Enrichment in DHA-Treated CRC Cells

To explore the signaling pathways underlying the anti-CRC effects of DHA, we conducted KEGG pathway enrichment analysis on differentially expressed genes (DEGs) identified in HCT116 cells following treatment with 20 μM or 50 μM DHA. This analysis identified five key signaling pathways that may be involved in DHA’s mechanism of action (Table 2).

Global KEGG/GO enrichment analyses showed that most pathways had an adjusted *p*-value (Q-value) > 0.05 after multiple testing correction, suggesting no statistically significant enrichment. However, multiple core genes related to the five signaling pathways were clustered in corresponding functional modules, suggesting the potential biological relevance of these pathways in DHA-mediated cytotoxicity, thus warranting cautious and targeted interpretation (Table 2).

#### 3.7.1. MAPK (Mitogen-Activated Protein Kinase) Signaling Pathway

**Figure 11:**
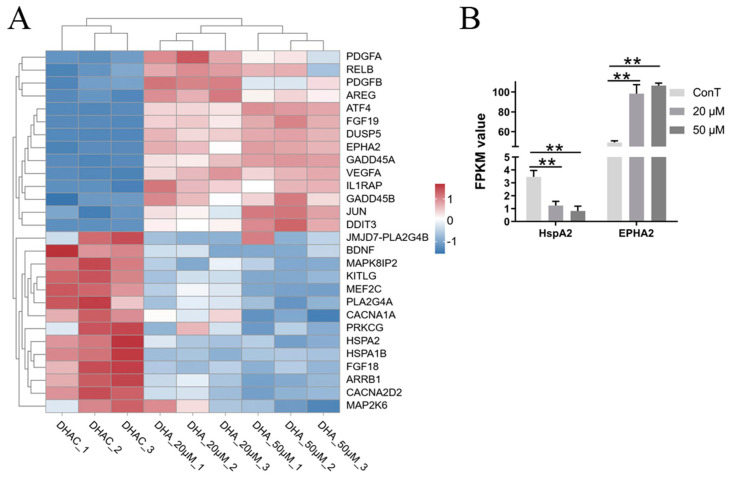
(**A**) Hierarchical clustering of MAPK pathway-associated DEGs. Color scale: blue, downregulated gene expression; red, upregulated gene expression; white, no change. (**B**) Transcriptome expression levels of genes related to the MAPK pathway. Data are presented as mean ± SD from *n* = 3 independent biological replicates. Statistical analysis was performed using two-way ANOVA compared to the control group. ** *p* < 0.01.

Transcriptomic analysis revealed that 20 μM DHA treatment yielded 18 DEGs, whereas 50 μM DHA treatment resulted in 23 DEGs (Figure 11A). This suggests that a higher dose of DHA elicits a stronger effect on the MAPK signaling pathway in HCT116 cells.

The MAPK pathway plays a crucial role in cellular responses to diverse external stimuli—including growth factors, inflammatory cytokines, and environmental changes—and is integral to intercellular communication. Evolutionarily conserved, this signaling cascade operates through a series of sequential phosphorylation events that, upon activation, elicit specific cellular outcomes [45]. Ephrin type-A receptor 2 (EPHA2), a receptor tyrosine kinase, is phosphorylated in a MAPK-dependent manner, thereby promoting tumor cell proliferation, migration, invasion, and angiogenesis, serving as a key driver of cancer progression [46]. Heat shock protein A2 (HSPA2) regulates the proliferation of lung adenocarcinoma cells via ERK1/2, a key subfamily of the MAPK cascade, and is significantly upregulated in lung adenocarcinoma tissues and cell lines [47]. DHA administration at concentrations of 20 and 50 μM both led to the downregulation of *EPHA2* and *HSPA2* expression (Figure 11B). While this suppressive effect lacked obvious dose dependence, DHA nonetheless impeded the malignant progression of CRC through this regulatory mode. Meanwhile, GADD45 acts as an upstream modulator of MAPK signaling [19]. In our study, DHA treatment (20 and 50 μM) upregulated the expression of *GADD45A* and *GADD45B* (Figure 4A), ultimately suppressing tumor growth.

#### 3.7.2. PI3K-Akt Pathway

**Figure 12:**
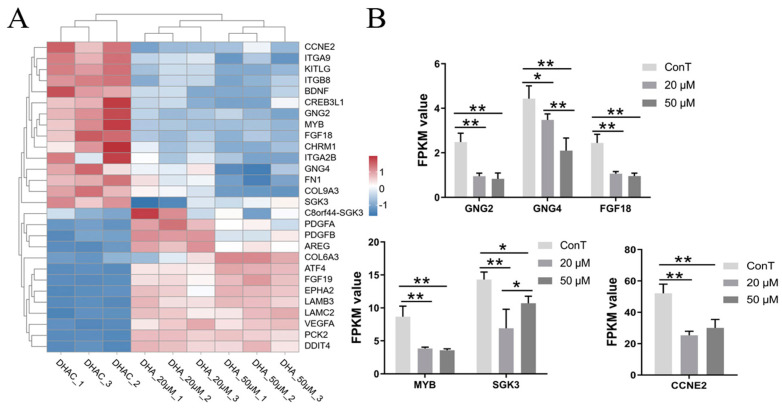
(**A**) Hierarchical clustering of PI3K/AKT pathway-associated DEGs. Color scale: blue, downregulated gene expression; red, upregulated gene expression; white, no change; (**B**) Transcriptome expression levels of genes related to the pathway. Data are presented as mean ± SD from *n* = 3 independent biological replicates. Statistical analysis was performed using one-way ANOVA followed by Tukey’s post hoc test and two-way ANOVA compared to the control group. * *p* < 0.05, ** *p* < 0.01.

The transcriptomic analysis revealed that treatment with 20 μM DHA yielded 18 DEGs, while 50 μM DHA treatment produced 22 DEGs (Figure 12A). These results suggest that a higher dose of DHA exert a more pronounced influence on the PI3K-Akt signaling pathway in HCT116 cells.

The PI3K/AKT signaling pathway was investigated given its well-established role in promoting tumor growth, survival, and therapy resistance in colorectal cancer (CRC). This pathway is frequently dysregulated in CRC and contributes to an aggressive phenotype and poor clinical outcomes [48]. G proteins are crucial for cellular signal transduction, relaying extracellular signals into the cell to regulate diverse biological processes. Activation of the PI3K-AKT pathway is often initiated by G protein-coupled receptors (GPCRs), which promote tumor invasion and metastasis by remodeling the cytoskeleton and activating Rho GTPases. Heterotrimeric G proteins consist of α, β, and γ subunits. The Gβγ dimer, in particular, participates in GPCR signaling and can activate effectors such as PI3Kγ, Ras, and MAPKs, thereby contributing to oncogenic signaling in cancer cells [49]. Both 20 and 50 μM DHA inhibited the expression of *GNG2* and *GNG4* (encoding Gγ2 and Gγ4), with the suppression of GNG4 showing a dose-dependent pattern (Figure 12B). Fibroblast growth factor 18 (FGF18) modulates cellular behavior by binding to its receptors and activating downstream signaling pathways. In cancer, aberrant FGF18 expression can drive uncontrolled proliferation, enhance angiogenesis, and remodel the tumor microenvironment, all of which contribute to disease progression. Notably, in clear cell renal cell carcinoma, FGF18 overexpression has been reported to suppress the PI3K/Akt pathway, highlighting its context-dependent role in oncogenic signaling [50]. In our results, DHA at 20 μM or 50 μM decreased *FGF18* expression levels with no obvious dose dependence (Figure 12B).

The transcription factor MYB has been implicated in activating oncogenic pathways, including PI3K/AKT. Aberrant MYB expression is closely associated with the development and progression of various malignancies, where it regulates tumor cell proliferation and differentiation [51]. Our results show that only a high dose of DHA (50 μM) downregulated *MYB* expression, suggesting a mechanism through which DHA may interfere with PI3K/AKT-driven oncogenic signaling in CRC. Serine/threonine-protein kinase 3 (SGK3), a serum- and glucocorticoid-regulated kinase, belongs to the AGC kinase family (which includes PKA, PKG, and PKC). SGK3 is dysregulated in various cancers and mediates tumorigenic processes, in part through the PI3K/AKT signaling pathway [52]. Transcriptomic results indicated that *SGK3* gene expression was dose-dependently downregulated by DHA treatment.

Cyclins are key regulators of the cell cycle. Dysregulation at any stage of the cycle can lead to cell cycle arrest and is frequently associated with cancer development. Cyclin E2 (CCNE2) plays a pivotal role in cell cycle control, primarily by promoting the transition from the G1 to S phase. Previous studies have demonstrated that CCNE2 participates in the PI3K/AKT pathway and contributes to cancer progression; for example, its inhibition suppresses non-small cell lung cancer [53,54]. The results indicated that DHA treatment at 20 μM or 50 μM downregulated *CCNE2* expression, and this suppressive effect did not show a distinct dose dependence (Figure 12B).

#### 3.7.3. Wnt Signaling Pathway

**Figure 13:**
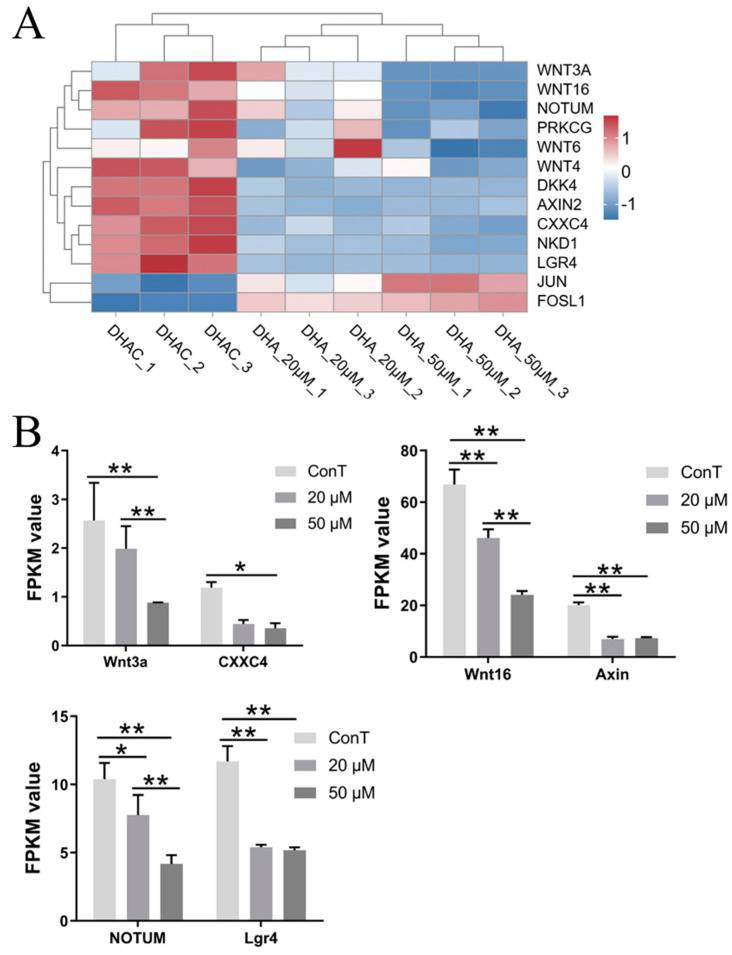
(**A**) Hierarchical clustering analysis of DEGs in the Wnt pathway. Color scale: blue, downregulated gene expression; red, upregulated gene expression; white, no change; (**B**) Transcriptome expression levels of genes related to the pathway. Data are presented as mean ± SD from *n* = 3 independent biological replicates. Statistical analysis was performed using two-way ANOVA compared to the control group. * *p* < 0.05, ** *p* < 0.01.

Transcriptomic profiling of this signaling pathway showed that treatment of HCT116 cells with 20 μM DHA resulted in 6 DEGs, whereas 50 μM DHA yielded 13 DEGs (Figure 13A). This demonstrates that increasing DHA doses elicits a more robust effect on the Wnt signaling pathway in HCT116 cells.

The Wnt signaling pathway plays a crucial regulatory role in cancer initiation and progression. It is frequently aberrantly activated in various cancers, leading to the upregulation of oncogenes. Axin is a tumor suppressor that acts as a scaffold protein and participates in multiple signaling pathways, such as Wnt and TGF-β, to regulate critical cellular processes including growth, proliferation, and carcinogenesis [55]. CXXC4, also known as IDAX, binds DNA and participates in the regulation of gene expression. It may influence cell proliferation and differentiation by modulating key components of the Wnt signaling pathway. Aberrant expression of CXXC4 is closely associated with the development and progression of various cancers [56]. Our findings reveal that DHA downregulated the expression of *Wnt3a*, *Wnt16*, *Axin*, and *CXXC4*, all of which were implicated in the malignant progression of colorectal tumors, thus supporting their potential as promising therapeutic targets for colorectal cancer. Notably, DHA-mediated suppression of *Wnt3a* and *Axin* lacked a distinct dose-dependent pattern, whereas its inhibitory effect on Wnt16 was dose-dependent. Additionally, a low DHA dose exerted no significant regulatory effect on *CXXC4* expression (Figure 13B).

NOTUM (formerly known as LOC147111) encodes a specialized glycosylphosphatidylinositol (GPI)-specific phospholipase that belongs to the α/β hydrolase family. By cleaving the GPI anchor, NOTUM inhibits Wnt signal transduction and thus acts as a negative regulator within the Wnt pathway [57]. In this study, DHA significantly suppressed the levels of *NOTUM*, thereby inhibiting tumor growth and metastasis. Notably, the inhibition of *NOTUM* showed dose-dependent effects in response to DHA treatment. Lgr4, a member of the G protein-coupled receptor family, is involved in the Wnt signaling pathway and plays a significant role in the development of various organs. It is highly expressed in gastrointestinal tumors and has been proposed as a potential biomarker for diagnosis, differential diagnosis, and prognosis assessment of these malignancies [58]. Our results demonstrate that DHA inhibited the expression of *Lgr4*, an effect that did not exhibit dose dependence (Figure 13B).

#### 3.7.4. TGF-β Signaling Pathway

Transcriptomic analysis of this signaling pathway demonstrated that treatment with 20 μM DHA induced 3 DEGs. In contrast, exposure to 50 μM DHA led to 8 DEGs. These results indicated that a higher dose of DHA exerted a more pronounced regulatory effect on the TGF-β signaling pathway in HCT116 cells.

**Figure 14:**
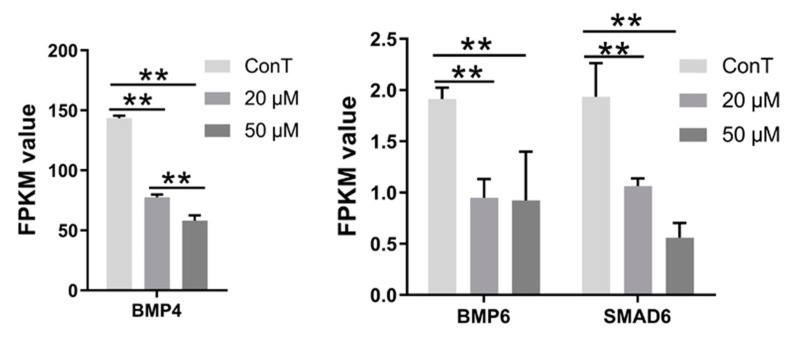
Transcriptome expression levels of genes related to the pathway. Data are presented as mean ± SD from *n* = 3 independent biological replicates. Statistical analysis was performed using one-way ANOVA followed by Tukey’s post hoc test and two-way ANOVA compared to the control group. ** *p* < 0.01.

The transforming growth factor-β (TGF-β) superfamily plays a pivotal role in development and tissue homeostasis by regulating the maintenance and regeneration of mature tissues. Members of this family range from multifunctional cytokines, such as TGF-β and bone morphogenetic proteins (BMPs), to factors with more specialized roles, such as anti-Müllerian hormone (AMH) and growth differentiation factor 8 (GDF8). These ligands regulate diverse cellular processes, including proliferation, differentiation, apoptosis, migration, and metabolism. As part of the TGF-β superfamily, BMPs can promote breast cancer cell invasion and metastasis by activating epithelial–mesenchymal transition (EMT) [59,60]. Furthermore, the canonical TGF-β signaling pathway is mediated by SMAD proteins, which, upon activation by receptor serine/threonine kinases, regulate cancer cell proliferation, differentiation, and death [61]. Our transcriptomic data revealed that *BMP4/6* and *SMAD6* were overexpressed in HCT116 cells. DHA treatment suppressed the expression of *BMP4/6* and *SMAD6*, thereby exerting an anti-cancer effect. Notably, the inhibition of *BMP4* by DHA exhibited a dose-dependent nature, whereas the suppression of *BMP6* and *SMAD6* did not differ significantly between the two doses (Figure 14).

#### 3.7.5. MicroRNAs Pathway

**Figure 15:**
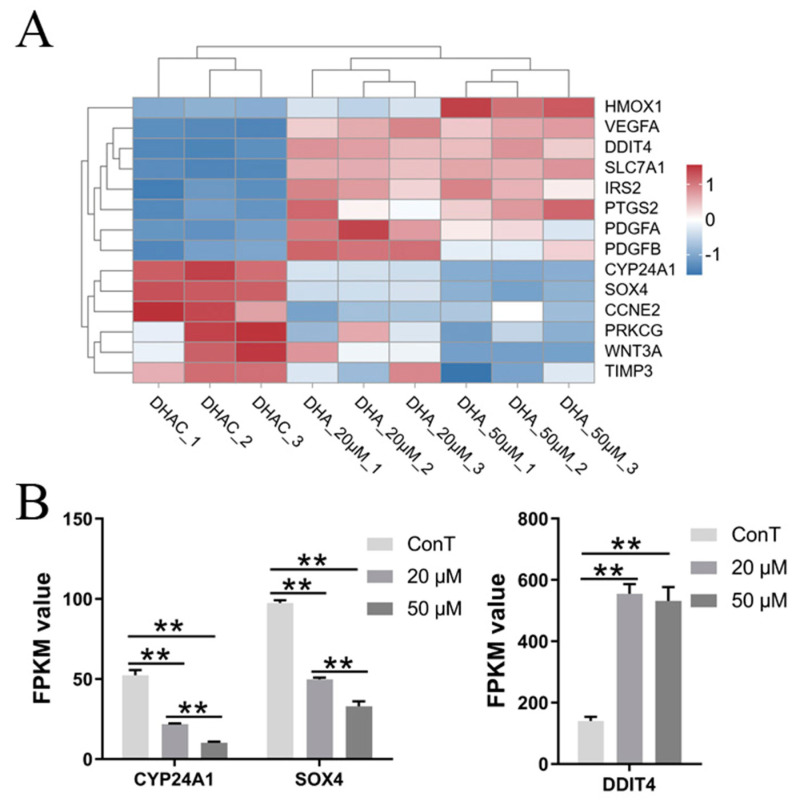
(**A**) Hierarchical clustering analysis of DEGs in the MicroRNAs pathway. Color scale: blue, downregulated gene expression; red, upregulated gene expression; white, no change. (**B**) Transcriptome expression levels of genes related to the pathway. Statistical analysis was performed using one-way ANOVA followed by Tukey’s post hoc test and two-way ANOVA compared to the control group. ** *p* < 0.01.

The transcriptome analysis of this signaling pathway revealed that treatment with 20 μM DHA resulted in 9 DEGs, whereas treatment with 50 μM DHA led to 11 DEGs (Figure 15A). This suggests that a higher dose of DHA has a more pronounced effect on the microRNA signaling pathway in HCT116 cells.

MicroRNAs (miRNAs) are a novel type of gene regulatory factor. They inhibit the expression of target genes or protein translation at the post-transcriptional level, thereby participating in the regulation of various physiological and pathological processes in the body, such as cell differentiation, proliferation, apoptosis, migration, and invasion [62]. miR-125b functions as a tumor suppressor and is downregulated in several malignancies, including cutaneous squamous cell carcinoma, oral squamous cell carcinoma, melanoma, and liver cancer. It exhibits significant tissue-specific effects on biological processes such as tumor cell proliferation and apoptosis [63]. Cytochrome P450 family 24 subfamily A member 1 (CYP24A1), a key enzyme in vitamin D metabolism, is frequently overexpressed in esophageal and CRCs with dysregulated miR-125b levels. This aberrant activation of CYP24A1 attenuates the anti-proliferative and pro-apoptotic effects of 1,25(OH)_2_D_3_ [64]. The results suggested that DHA inhibited the expression of the *CYP24A1* gene through miR-125b, thereby blocking cancer progression, and this effect was dose-dependent (Figure 15B). Studies have shown that miR-221 is aberrantly expressed in multiple tumor types. The DNA damage-inducible transcription factor 4 (DDIT4), a direct and bona fide target of miR-221, plays a key role in inducing cell cycle arrest, inhibiting cell growth, and regulating cell death [65]. In the present study, we found that DHA upregulated *DDIT4* expression independently by modulating miR-221, thereby suppressing the progression of CRC (Figure 15B). The transcription factor SOX4 (SRY-related high-mobility-group box 4) is frequently overexpressed in various malignancies and contributes to tumor initiation and progression. It has been shown to inhibit apoptosis, promote cell invasion and metastasis, and play a role in the induction and maintenance of cancer precursor cells [66]. Given that SOX4 is a well-established target of miR-335, our transcriptomic analysis revealed that DHA downregulated *SOX4* expression via miR-335, thereby suppressing CRC progression in a dose-dependent manner (Figure 15B).

MicroRNA-targeted therapeutic approaches, encompassing miRNA mimics and inhibitory agents, exhibit considerable translational potential for clinical cancer intervention. Nevertheless, several key hurdles remain to be overcome, notably the low efficiency of drug delivery systems and the profound heterogeneity of tumor tissues. Future research efforts should focus on further clarifying the complex microRNA regulatory networks across diverse cancer subtypes and developing optimized combination therapeutic strategies to enhance clinical efficacy and applicability.

## 4. Discussion

This study integrated RNA-seq-based transcriptomic profiling and functional validation to dissect the DEG-mediated dose-specific regulatory networks underlying the anti-tumor activity of DHA in CRC HCT116 cells. By focusing on differentially expressed genes (DEGs) and their orchestration of molecular pathways, our findings provided novel genome-centric insights into DHA’s therapeutic potential for colorectal cancer (CRC), aligning with our core focus of gene regulatory mechanisms and transcriptional networks.

First, MTT assays confirmed that DHA inhibits HCT116 cell viability in a dose-dependent manner (IC_50_ = 50 μM), consistent with prior reports of DHA’s dose-dependent cytotoxicity in pancreatic (50 μM-induced apoptosis) and lung cancers (IC_50_: 20.5–51.2 μM) [10,11]. Notably, low-dose DHA (20 μM) reduced viability by 20–30% and induced 280 DEGs, whereas high-dose DHA (50 μM) exerted a more potent inhibitory effect accompanied by 678 DEGs—representing a ~2.4-fold expansion of transcriptional perturbations. This dose-dependent expansion of the DEG landscape reflected a threshold-dependent transcriptional reprogramming event, wherein CRC cells required sufficient DHA exposure to trigger extensive gene expression rewiring. Such DEG heterogeneity underscored the existence of distinct dose-specific genetic programs that drove DHA’s anti-tumor effects, a key observation that reinforced the value of transcriptomic profiling in unraveling context-dependent drug mechanisms.

Functional enrichment analysis of DEGs revealed that DHA modulates multiple cell death pathways via gene-specific regulation—a hallmark of its anti-tumor activity. Low-dose DHA primarily orchestrated apoptosis through DEG-driven upregulation of *GADD45α/β* and activation of the *ATF4/DDIT3* endoplasmic reticulum stress (ERS) axis, wherein these DEGs served as upstream regulators of apoptotic signaling. In contrast, high-dose DHA converged on ferroptosis as a pivotal DEG-mediated mechanism, evidenced by transcriptional upregulation of *MAP1LC3B* (promoting autophagy-dependent iron release) and *HMOX1* (enhancing heme degradation and ferrous iron accumulation), alongside downregulation of *SLC40A1* (disrupting iron export) and *STEAP3* (impairing iron homeostasis). As an iron-dependent regulated cell death pathway with emerging relevance as a cancer therapeutic target, ferroptosis has not previously been linked to high-dose DHA in CRC—our study filled this gap by identifying ferroptosis-related DEGs as core mediators of DHA’s high-dose efficacy. Additionally, DHA promoted cellular senescence in a dose-dependent manner via DEG-driven activation of the *ATF4/DDIT3/TRIB3* pathway and upregulation of *GADD45α/β*: 20 μM DHA induced senescence after 7 days, while 50 μM DHA achieved comparable effects within 1 day (validated by SA-β-Gal staining). This senescence phenotype, driven by key DEGs, may contribute to long-term tumor suppression by arresting cancer cell proliferation at the transcriptional level.

Metabolic reprogramming, a critical hallmark of cancer, was another key DEG-mediated mechanism underlying DHA’s efficacy. Both doses downregulated metabolism-related DEGs encoding rate-limiting enzymes in glycolysis (*KHK*, *ALDOC*), lipid metabolism (*SCD1*, *HMGCS1*), and folate metabolism (*DHFR*)—directly impairing the metabolic adaptability that sustained tumor growth. A high-dose DHA further suppressed *MGAT5B*, a DEG encoding a key enzyme in N-glycan branching; this unique regulation of glycosylation-related DEGs is particularly relevant, as MGAT5B-mediated glycan modification promotes cancer cell adhesion and metastasis [44]. KEGG enrichment analysis corroborated that DEGs were significantly enriched in “metabolic processes,” highlighting DHA’s capacity to disrupt tumor metabolism via targeted regulation of metabolic DEGs—a finding that reinforces the role of transcriptional networks in shaping cancer metabolic phenotypes.

DEG-centered signaling pathway enrichment identified five core oncogenic pathways (MAPK, PI3K-Akt, Wnt, TGF-β, and microRNA regulatory networks) that were differentially targeted by DHA in a dose-dependent manner. Low-dose DHA repressed DEGs such as *EPHA2* (MAPK pathway) and *GNG2/4* (PI3K-Akt pathway), thereby attenuating pro-tumor signaling cascades. In contrast, high-dose DHA additionally inhibited the *Wnt* and *TGF-β* pathways via downregulation of DEGs including *Wnt3a/16* and *BMP4/6*—extending its regulatory scope to more oncogenic networks. The microRNA pathway also exhibited dose-specific DEG regulation: DHA modulated the miR-125b/CYP24A1, miR-221/DDIT4, and miR-335/SOX4 axes, wherein these DEG-driven miRNA-mRNA interactions directly regulated cancer cell proliferation and apoptosis [63,64]. This multi-pathway targeting via DEG networks indicated that DHA acts as a pleiotropic anti-tumor agent, potentially reducing therapeutic resistance by disrupting interconnected oncogenic gene regulatory cascades—a key advantage highlighted by transcriptomic profiling.

Several limitations of this study should be acknowledged. First, all experiments were performed only in the HCT116 colorectal cancer cell line. Further validation in other CRC cell lines with distinct genetic backgrounds (e.g., microsatellite instability-high vs. microsatellite stable) and in vivo models is warranted to confirm the generalizability of DEG-mediated regulatory networks. Furthermore, although our transcriptomic analysis identified multiple ferroptosis-related genes involved in DHA-induced cell death, further verification using ferroptosis inhibitors, direct detection of lipid peroxidation, intracellular iron levels, and other typical ferroptotic markers are warranted in future research to consolidate the mechanistic conclusion. In addition, the specific crosstalk between DEG-driven signaling pathways and cell death mechanisms (e.g., how ferroptosis-related DEGs interact with apoptotic or senescence-associated gene networks) merit further investigation via co-expression analysis or gene silencing/overexpression assays.

## 5. Conclusions

In conclusion, this study established a genome-centric framework for understanding DHA’s dose-dependent anti-cancer activity in CRC: low-dose DHA primarily drove apoptosis and senescence via a restricted set of DEGs and core pathways (MAPK, PI3K-Akt, microRNAs), while high-dose DHA triggered ferroptosis and broader metabolic/glycosylation repression through an expanded DEG network that additionally targets Wnt and TGF-β pathways. These findings not only deepen our understanding of DHA’s DEG-mediated mode of action but also provide novel gene targets (e.g., *HMOX1*, *SLC40A1*, *MGAT5B*) and a rational basis for optimizing DHA-based therapeutic regimens—for instance, low doses for long-term maintenance therapy (leveraging senescence/apoptosis) and high doses for targeted tumor suppression (prioritizing ferroptosis). Future research should focus on translating these in vitro DEG findings to clinical settings, exploring combination therapies that synergize with DHA to enhance DEG-driven anti-tumor effects, and developing gene-informed targeted delivery systems to improve DHA’s efficacy while minimizing off-target effects. Overall, our transcriptomic analysis uncovers dose-specific gene regulatory networks that govern DHA’s anti-CRC activity, offering valuable insights for advancing genome-guided cancer therapeutics.

## Figures and Tables

**Table 1:** Overview of the sequencing reads from the different doses of DHA treatment with HCT116 cells.

Sample	Raw Reads	Q30 (%)	GC (%)	Clean Reads	Q30 (%)	GC (%)	Mapped Clean Reads (%)
Control (0 μM DHA)							
H116_DHAC_1	44,188,712	93.39	45.81	43,986,358	93.72	45.64	96.26
H116_DHAC_2	42,483,880	94.34	45.85	42,374,856	94.59	45.77	96.01
H116_DHAC_3	48,602,680	94.32	46.55	48,447,860	94.76	46.42	95.91
Low-dose (20 μM DHA)							
H116DHA1-1	50,915,624	93.24	46.64	50,703,850	93.77	46.50	94.69
H116DHA1-2	47,704,438	94.80	46.47	47,566,216	95.18	46.35	96.29
H116DHA1-3	42,442,182	95.23	45.83	42,347,122	95.51	45.72	96.30
High-dose (50 μM DHA)							
H116DHA2-1	44,217,258	94.52	46.05	44,118,238	94.81	45.93	96.45
H116DHA2-2	42,637,164	94.27	45.91	42,538,072	94.51	45.82	96.26
H116DHA2-3	45,737,250	93.67	45.47	45,580,986	94.06	45.35	96.48

**Table 2:** The key Signaling Pathway Enrichment in DHA-Treated CRC Cells.

Pathway	Pathway ID	*p*-Value/Q-Value (H116_DHAC vs. H116DHA1)	*p*-Value/Q-Value (H116_DHAC vs. H116DHA2)
MAPK signaling pathway	ko04010	0.000027/0.006758	0.001052/0.062093
PI3K-Akt signaling pathway	ko04151	0.000821/0.101972	0.019604/0.275392
Wnt signaling pathway	ko04310	0.107381/0.483097	0.009313/0.196228
TGF-beta signaling pathway	ko04350	0.298999/0.663568	0.029361/0.320792
MicroRNAs in cancer	ko05206	0.005445/0.137767	0.048812/0.375047

## Data Availability

The original contributions presented in this study are included in the article/Supplementary Materials. Further inquiries can be directed to the corresponding author(s).

## Supplementary Materials

The following supporting information can be downloaded at: https://www.mdpi.com/article/10.3390/cimb48040342/s1.
