# Supplementary material for: Dihydroartemisinin Unravels Dose-Dependent Transcriptomic Networks Orchestrating Ferroptosis and Metabolic Reprogramming in Colorectal Cancer
Source: Curr Issues Mol Biol. 2026 Mar 25;48(4):342. doi: 10.3390/cimb48040342 (PMC13115041; doi:10.3390/cimb48040342)
Supplement: Supplementary file 1 [file cimb-48-00342-s001.zip › cimb-4191586-supplementary.pdf]

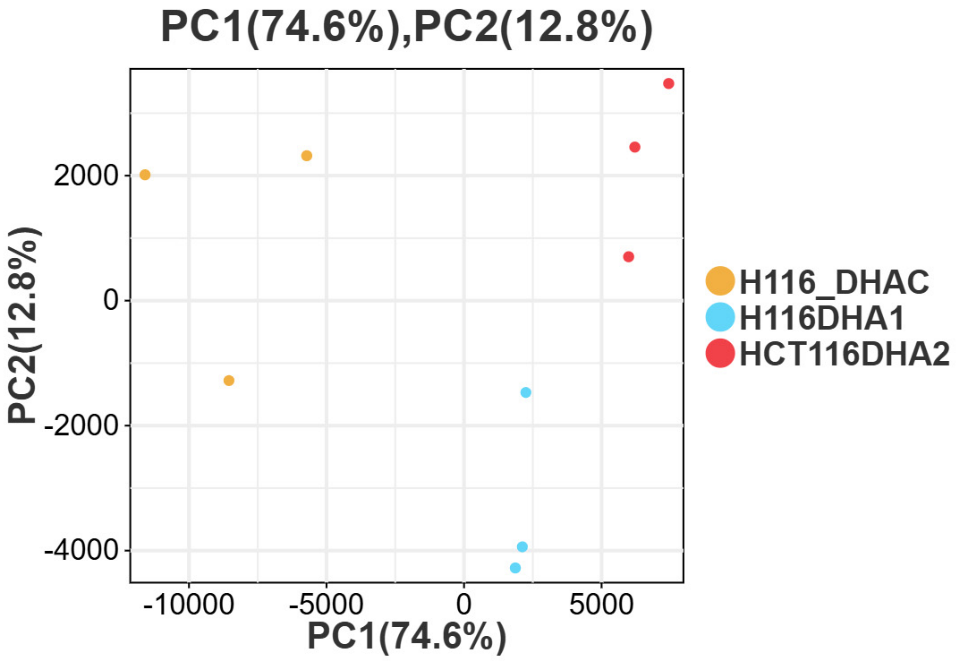

Figure S1. Principal component analysis (PCA) of transcriptomic profiles in HCT116 cells treated with DHA.

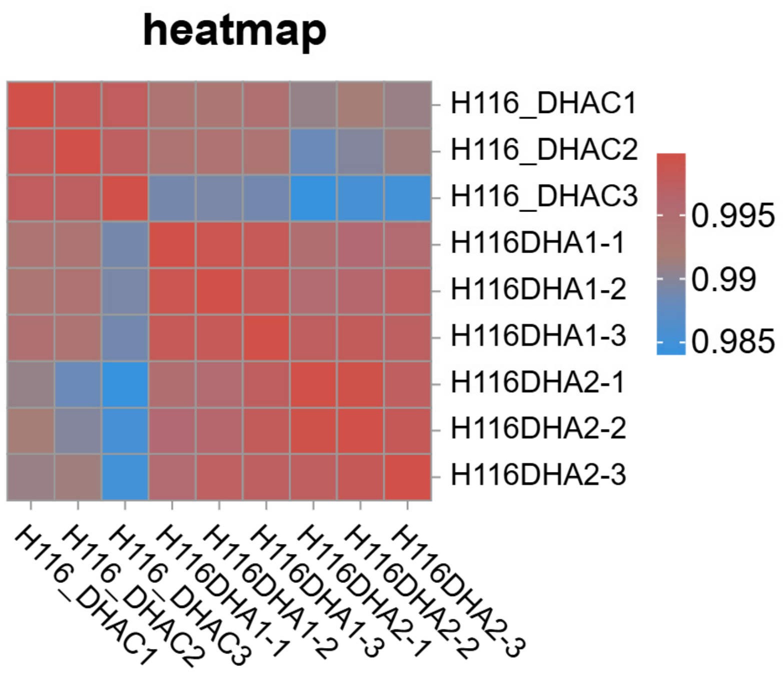

Figure S2. Correlation heatmap of HCT116 cell transcriptomic samples.

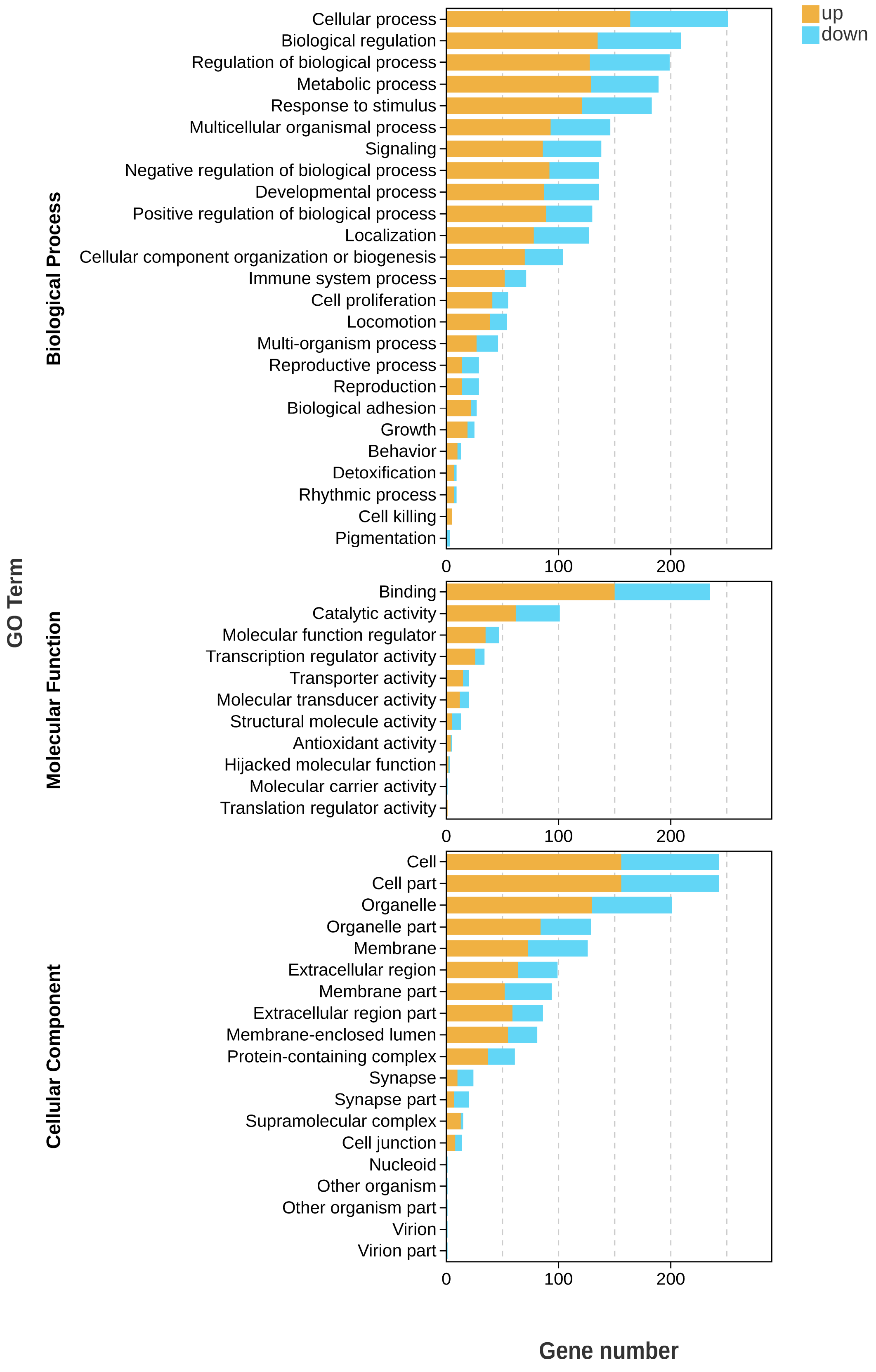

Figure S3. GO classification of DEGs in DHA (20 μM)-treated HCT116 cells.

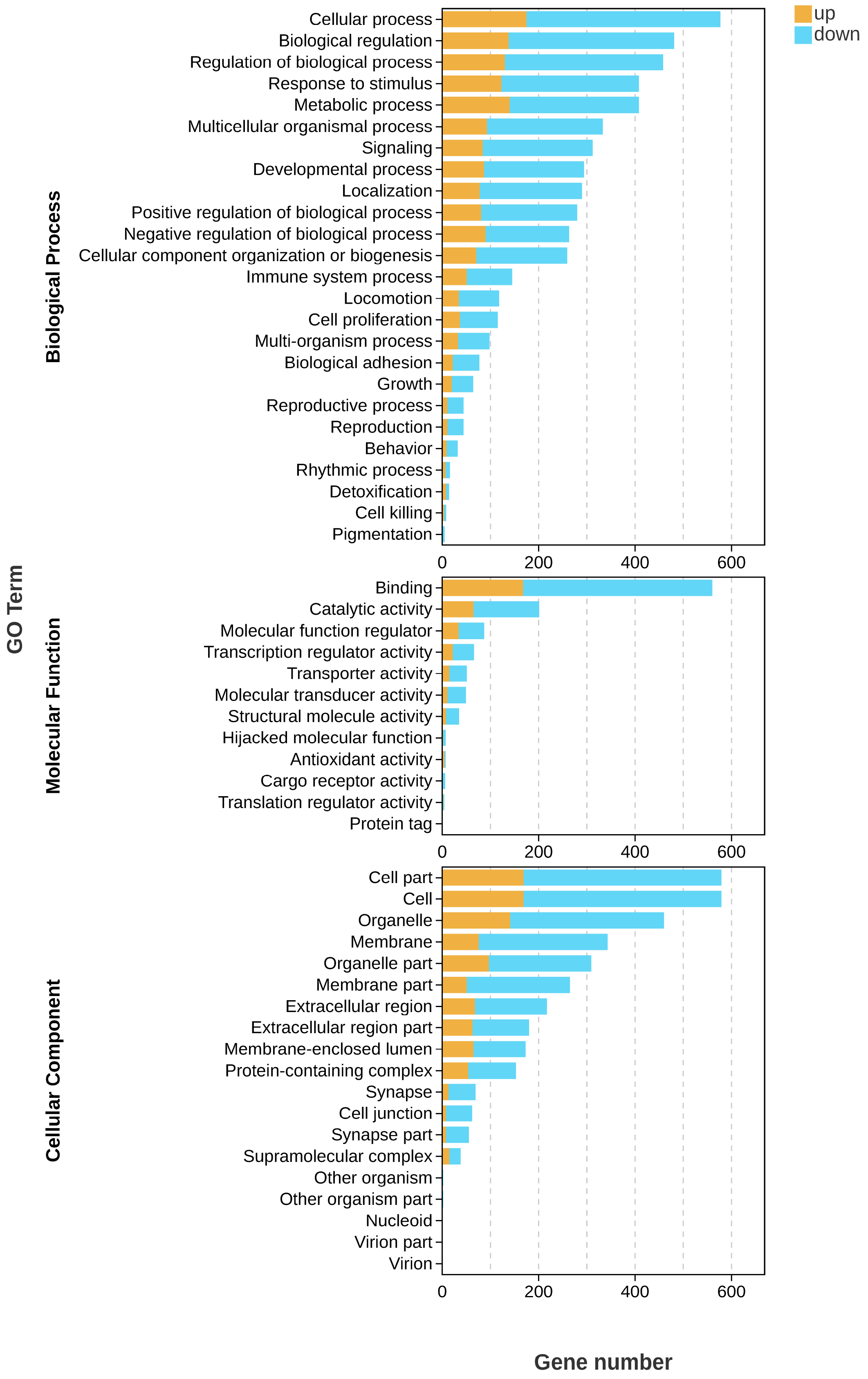

Figure S4. GO classification of DEGs in DHA (50 μM)-treated HCT116 cells.

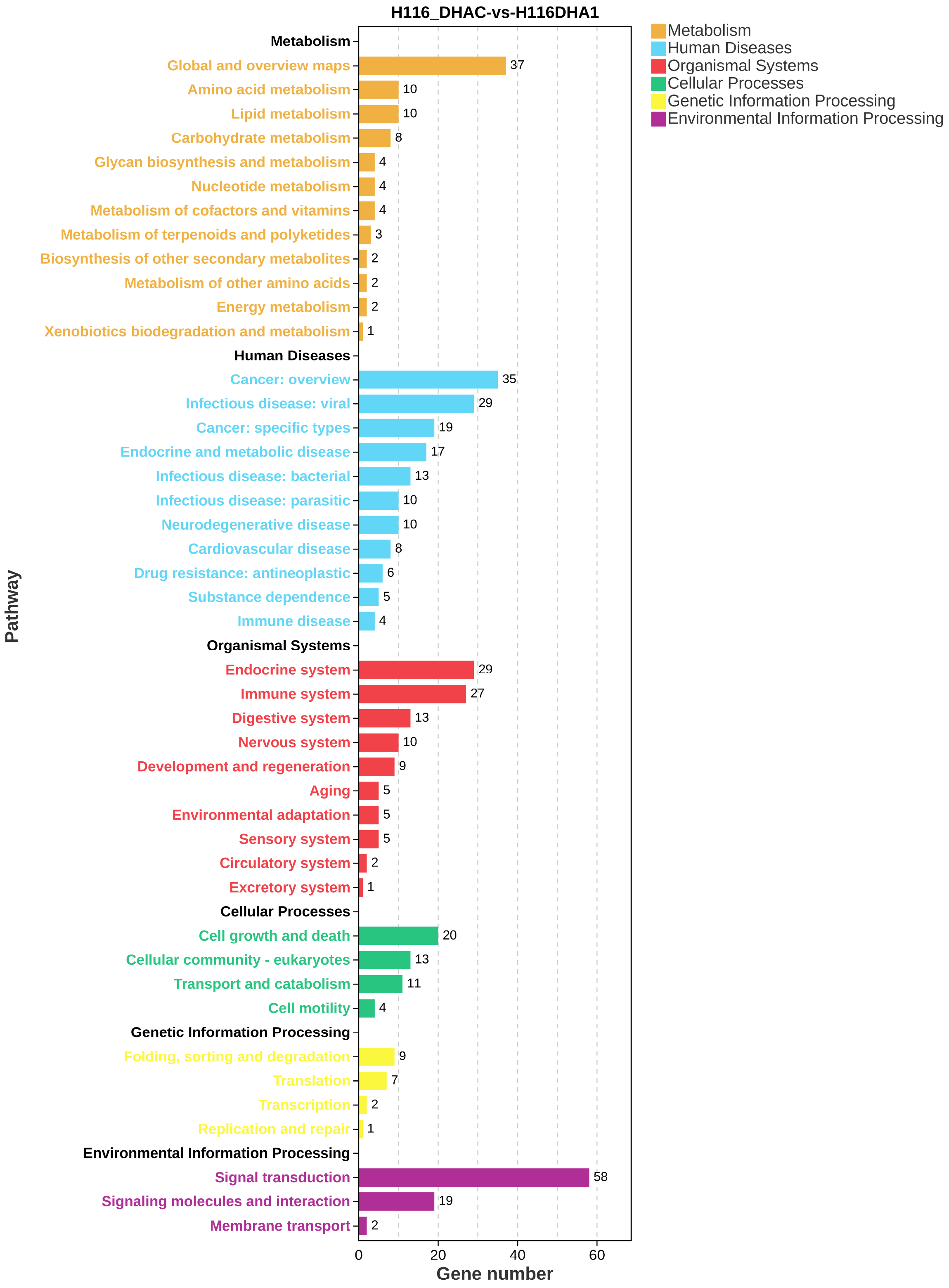

Figure S5. KEGG pathway enrichment analysis of DEGs in DHA (20 μM)-treated HCT116 cells.

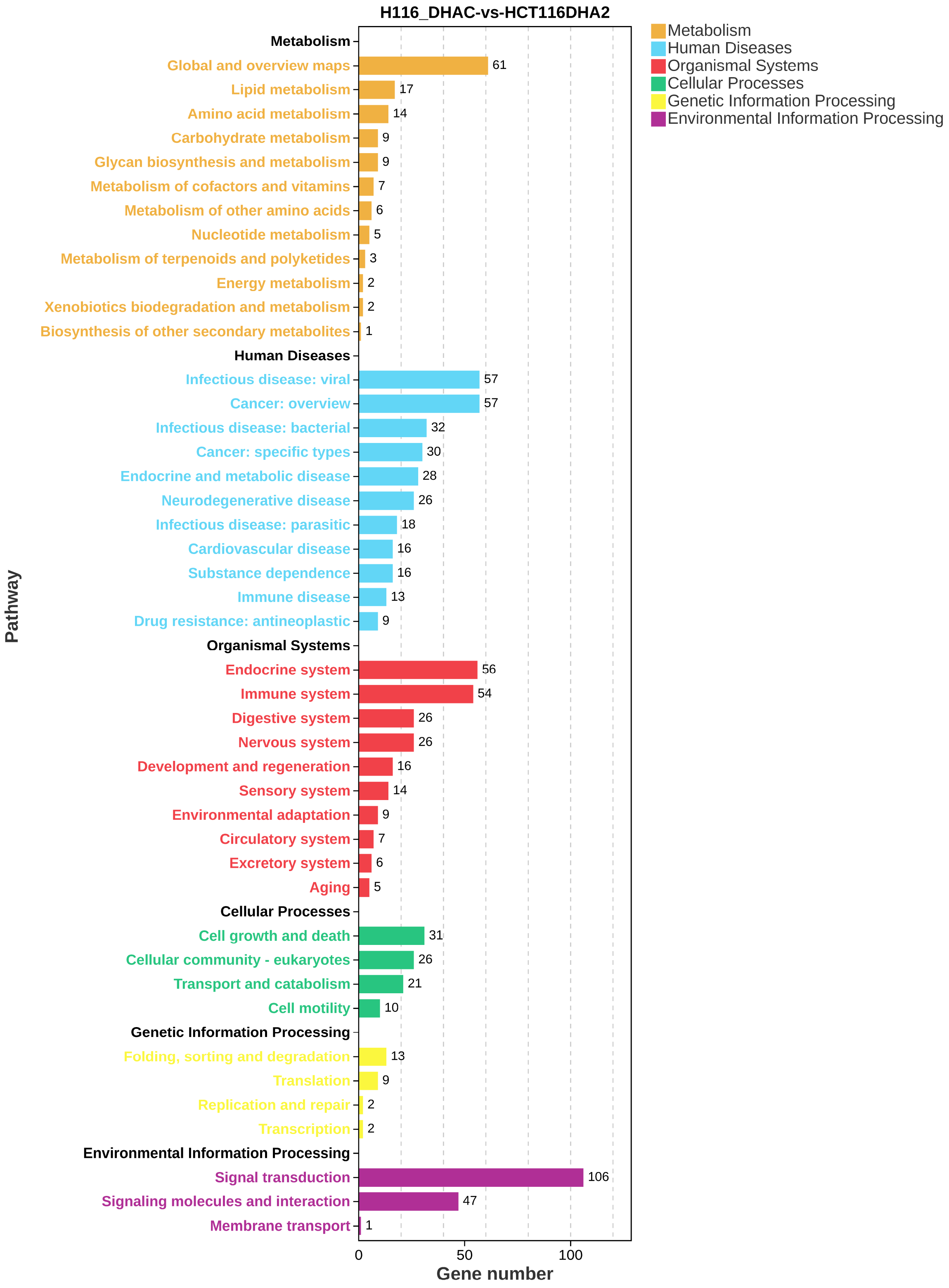

Figure S6. KEGG pathway enrichment analysis of DEGs in DHA (50 μM)-treated HCT116 cells.
